# Localizing Ashkenazic Jews to Primeval Villages in the Ancient Iranian Lands of Ashkenaz

**DOI:** 10.1093/gbe/evw046

**Published:** 2016-03-03

**Authors:** Ranajit Das, Paul Wexler, Mehdi Pirooznia, Eran Elhaik

**Affiliations:** ^1^Department of Animal and Plant Sciences, University of Sheffield, Sheffield, UK; ^2^Manipal Centre for Natural Sciences (MCNS), Manipal University, Manipal, Karnataka, India; ^3^Department of Linguistics, Tel Aviv University, Tel-Aviv, Israel; ^4^Department of Psychiatry and Behavioral Sciences, Johns Hopkins University

**Keywords:** archaeogenetics, Yiddish, Ashkenazic Jews, Ashkenaz, geographic population structure (GPS), Rhineland Hypothesis

## Abstract

The Yiddish language is over 1,000 years old and incorporates German, Slavic, and Hebrew elements. The prevalent view claims Yiddish has a German origin, whereas the opposing view posits a Slavic origin with strong Iranian and weak Turkic substrata. One of the major difficulties in deciding between these hypotheses is the unknown geographical origin of Yiddish speaking Ashkenazic Jews (AJs). An analysis of 393 Ashkenazic, Iranian, and mountain Jews and over 600 non-Jewish genomes demonstrated that Greeks, Romans, Iranians, and Turks exhibit the highest genetic similarity with AJs. The Geographic Population Structure analysis localized most AJs along major primeval trade routes in northeastern Turkey adjacent to primeval villages with names that may be derived from “Ashkenaz.” Iranian and mountain Jews were localized along trade routes on the Turkey’s eastern border. Loss of maternal haplogroups was evident in non-Yiddish speaking AJs. Our results suggest that AJs originated from a Slavo-Iranian confederation, which the Jews call “Ashkenazic” (i.e., “Scythian”), though these Jews probably spoke Persian and/or Ossete. This is compatible with linguistic evidence suggesting that Yiddish is a Slavic language created by Irano-Turko-Slavic Jewish merchants along the Silk Roads as a cryptic trade language, spoken only by its originators to gain an advantage in trade. Later, in the 9th century, Yiddish underwent relexification by adopting a new vocabulary that consists of a minority of German and Hebrew and a majority of newly coined Germanoid and Hebroid elements that replaced most of the original Eastern Slavic and Sorbian vocabularies, while keeping the original grammars intact.

## Introduction

Paramount geographical movements, due to voluntary migration or forced resettlement, are often reflected in a language’s lexicon as a new stratum of words and phrases that may replace or modify archaic terms. In an analogy to species’ struggle to survive, Darwin remarked that “a struggle for life is constantly going on among the words and grammatical forms in each language” (1871). This parallelism between the history of a language and its speakers and the expectation that such insights will highlight the geographical origins of populations have attracted much attention from geneticists and linguists (Cavalli-Sforza 1997; Kitchen et al. 2009; Balanovsky et al. 2011; Bouckaert et al. 2012). Major deviations from this parallelism are explicable by admixture or migration followed by extreme isolation (Ramachandran et al. 2005). In such cases, the language’s lexicon may represent various strata of words from different languages the migrating people have encountered, deeming most phylogenetic-based approaches inapplicable. For that reason, it has been proposed to look at linguistic and genetic data in parallel and attempt integrative analyses (Brandt et al. 2014).

One of the last European languages whose linguistic and geographical classifications remain unclear even after three centuries of research is Slavic Yiddish (Weinreich 2008), the native language of the Ashkenazic Jewish community, whose own origins is still under debate (e.g., Costa et al. 2013; Elhaik 2013). The Slavic Yiddish (now called universally simply Yiddish), spoken since the 9th century, consists of Hebrew, German, Slavic, and other elements written in Aramaic characters (Weinreich 2008). Because of its many radical deviations from native German norms, its alleged cognate language, Yiddish has been rudely labeled both by native and nonnative speakers as “bad German” and in Slavic languages as a “jargon” (Weinreich 2008). Part of the problem in deciphering its origin is that over the centuries Yiddish speakers have invented a huge number of “Germanoid” (German-like) and “Hebroid” (Hebrew-like) components coined by nonnative speakers of those languages based on Slavic or Iranian models alongside authentic Semitic Hebrew and German components. An example of an invented phrase is Modern Hebrew *paxot o joter* (literally “less or more”) that imitates the same written Ashkenazic Hebroid phrase, derived from Upper Sorbian and Iranian languages, but not Old Semitic Hebrew. The overwhelming majority of the world’s languages use “more or less.” This expression appeared during the Middle Ages, long after the death of spoken Hebrew and possibly a millennium before the appearance of modern-day “Modern Hebroid” (= Israeli Hebrew). These and other invented features made the components of Yiddish word strata and their relationship to other languages multilayered, porous, fugacious, and difficult to localize.

**Fig. 1.—:**
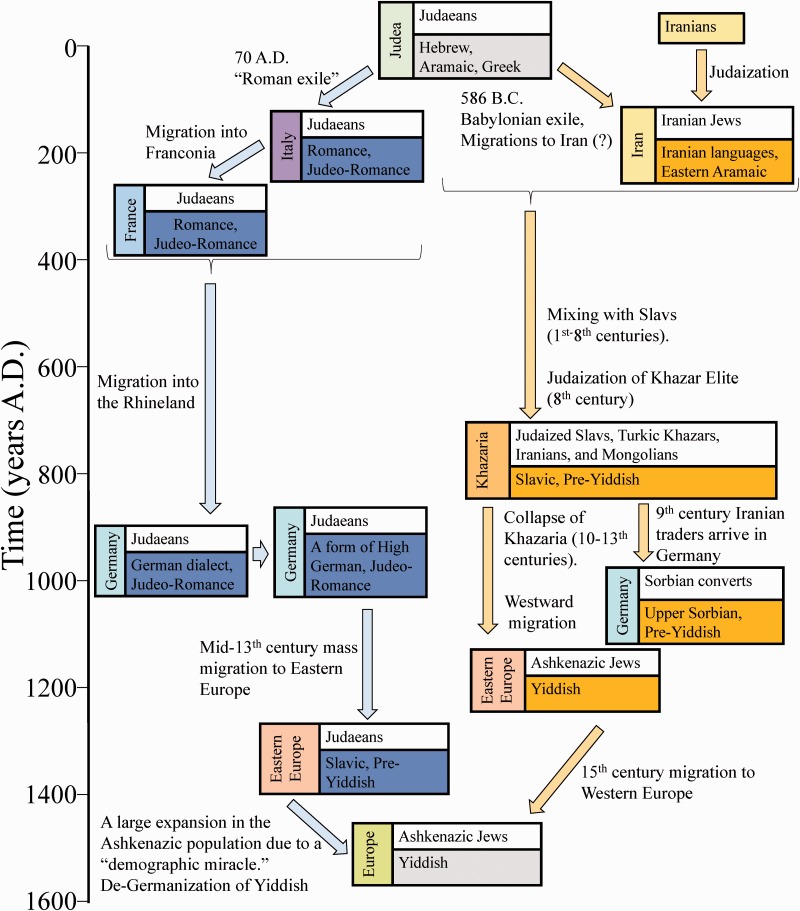
An illustrated timeline for the events comprised by the Rhineland (blue arrows) and the Irano-Turko-Slavic (orange arrows) hypotheses. The stages of Yiddish evolution according to each hypothesis are shown through landmark events for which the identity of the proto-Ashkenazic Jewish populations and their spoken languages are noted per region.

The work of Cavalli-Sforza and other investigators have already established the strong relationship between geography, genetics, and languages (Cavalli-Sforza et al. 1994; Eller 1999; Balanovsky et al. 2011; Everett 2013), implying that the geographical origin of Yiddish would correspond to that of Yiddish speakers. However, the genomes of Yiddish speakers were never studied, and the admixed nature of both Yiddish (King 2001; Wexler 2010) and Ashkenazic Jewish genome (Bray et al. 2010; Elhaik 2013) preclude using traditional approaches to localize their geographical origins. It is also unclear whether AJ subgroups share common origins (Elhaik 2013). To improve our understanding about the geographical and ancestral origins of contemporary AJs, genome-wide and haplogroup analyses and comparison with Jewish and non-Jewish populations were performed. Our findings are evaluated in light of the two major linguistic hypotheses depicting a German or Turkic (Khazar), Ukrainian, and Sorbian (in the eastern German lands) geographical origins for Yiddish and AJs (table 1, fig. 1).

**Table 1:** Two Hypotheses Regarding the Origin of the Yiddish Language and Lexicography

**Hypotheses**	**Lexicographical admixture**	**Origins**	**References**
Rhineland	80% German, 15% Hebrew, and 5% Slavic	Southwestern (Rhineland) and Southeastern Germany (Bavaria)	King (2001) and Weinreich (2008)
Irano-Turko-Slavic	Slavic (43%), German and Germanoid (35%), Hebrew and Hebroid (8%), and the remaining (14%) are Iranian, Turkic and unique Romance, Arabic (including Berberized Arabic), and Greek	The Khazar’s Empire Kievan Rus' (today's Ukraine) Sorbian areas of Germany	Wexler (2010)

The Rhineland hypothesis differs from the Irano-Turko-Slavic hypothesis by ignoring the Iranian component alongside the “Hebroidisms” and “Germanoidisms,” whose geographical origins are unclear. Both hypotheses, however, agree on the same three basic components: German, Slavic, and Hebrew, though they disagree on their proportions.

The “Rhineland hypothesis” envisions modern Yiddish speaking AJs to be the descendants of the ancient Judaeans. The presence of Jews in Western and, later, Eastern Europe is explained, in an oversimplified manner, by two allegedly mass migratory waves, first from ancient Israel to Roman Empire, then later from what is now Germany to Slavic lands (van Straten and Snel 2006; Sand 2009). The theory posits the “Roman Exile” that followed the destruction of Herod’s temple (70 A.D.) as introducing a massive Jewish population to Roman lands (King 2001). Yiddish is assumed to have developed in the 9th to 10th century when Romance-speaking French and Italian Jews migrated to the Rhineland (and Franconia) and replaced their Romance speech with local German dialects (Weinreich 2008). The absence of local Rhineland German dialect features in Yiddish subsequently prompted linguists to relocate its birthplace to Bavaria (King 2001). It was these Jews who created the so-called Ashkenazic culture, named after the Medieval Hebrew term for the German lands. The second migration wave took place in the 13th century, when German Jews allegedly migrated into monolingual Slavic lands and rapidly reproduced via a “demographic miracle” (Ben-Sasson 1976).

The competing “Irano-Turko-Slavic” hypothesis considers AJs to be the descendants of a heterogeneous Iranian population, which later mixed with Eastern and Western Slavs and possibly some Turks and Greeks in the territory of the Khazar Empire around the 8th century A.D. The name “Ashkenaz” is the Biblical Hebrew adaptation of the Iranian tribal name, which was rendered in Assyrian and Babylonian documents of the 7th century B.C. as *aškūza*, called in English by the Greek equivalent “Scythian” (Wexler 2010). Already by the 1st century, most of the Jews in the world resided in the Iranian Empire (Baron 1952). These Jews were descended either from Judaean emigrants or, more likely, from local converts to Judaism and were extremely active in international trade, as evident from the Talmud and non-Jewish historical sources (Baron 1957; Gil 1974). Over time, many of them moved north to the Khazar Empire to expand their mercantile operations. Consequently, some of the Turkic Khazar rulers and the numerous Eastern Slavs in the Khazar Empire converted to Judaism to participate in the lucrative Silk Road trade between Germany and China (Foltz 1998), which was essentially a Jewish monopoly (Rabinowitz 1945, 1948; Baron 1957). Yiddish emerged at that time as a secret language for trade based on Slavic and even Iranian patterns of discourse. When these Jews began settling in Western and Eastern Slavic lands, Yiddish went through a relexification process, that is, replacing the Eastern Slavic and the newly acquired Sorbian vocabularies with a German vocabulary while keeping the original grammar and sound system intact (Wexler 2011a). Critics ofthis hypothesis cite the fragmentary and incomplete historical records from the first millennium (King 1992) and discount the relevance of relexification to Yiddish studies (Wexler 2011b).

Assuming the history of Yiddish and AJs is parallel (Weinreich 2008), at least in part, localizing the genomic admixture signature of Yiddish and non-Yiddish speaking AJs may also unveil the birthplaces of Yiddish and AJs, respectively. Due to the changes in the population structure of AJs over the past millennia, we do not expect our biogeographical predictions to perfectly agree with the predictions made by either hypothesis. This is the first study that analyzes genetic data of Yiddish speakers, and it is carried out at a most timely manner as individuals who speak solely Yiddish are increasingly difficult to find (Wallet 2006; Niborski 2009; Shin and Kominski 2010).

## Results

We analyzed the genomes of 367 public participants of the Genographic Project who reported having Ashkenazic Jewish parents. They were further subdivided to 186 descendants of sole Yiddish speakers (or “Yiddish speakers”) and 181 descendants of multi-lingual or non-Yiddish speakers (or “non-Yiddish speakers”). Country of residence was reported by 94% Yiddish and non-Yiddish speakers with the vast majority of all individuals living in the United States (table 2). We note that these figures do not correspond to the geographic distribution of Yiddish speakers and overrepresent the share of Americans (Shin and Kominski 2010) mainly at the expense of Ultra-Orthodox Jews, one of the largest group of Yiddish speakers (Isaacs 1998). However, since the parents of all the individuals studied here are Europeans, the sample bias probably reflects choices of contemporary residency rather than ancestral origins and is unlikely to have a large effect on our results.

**Table 2:** Modern-Day Residency of AJs in this Study

**Country**	**Yiddish speakers (*n* = 186) (%)**	**Non-Yiddish speakers (*n* = 181) (%)**
United States	90	82
Canada	4	3
Israel	2	3
United Kingdom	2	6
South Africa	1	0
Australia	1	2
Russia	1	0
Switzerland	1	0
Brazil	0	1
Chile	0	1
China	0	1
Norway	0	1
Puerto Rico	0	1

All biogeographical inferences were carried out using the geographic population structure (GPS) tool (Elhaik et al. 2014). In brief, GPS infers the geographical coordinates of an individual by matching its admixture proportions with those of reference populations known to reside in a certain geographical region for a substantial period of time. Whereas a population’s movement followed by gene exchanges with other populations modifies its admixture signature, isolation, and segregation preserve the original admixture signature of the migratory population. GPS predictions should therefore be interpreted as the last place that admixture has occurred, termed here *geographical origin*. For an individual of mixed origins, the inferred coordinates represent the mean geographical locations of their immediate ancestors.

Our search for the geographical origins of AJs was focused on Eurasia, with particular consideration of the area covering the regions predicted by each hypothesis (table 1, fig. 1). This area encompasses German lands, South Russia, and the area between ancient Judea and the western regions of the former Iranian (Sassanian) Empire. With the exception of a pre-Scythian Iron Age individual included in our analyses, the absence of sufficient ancient DNA from the relevant time period required using modern-day populations as substitutes may restrict our ability to ascertain all the founding populations of AJs.

### Biogeographical Mapping of Afro-Eurasian Populations

**Fig. 2.—:**
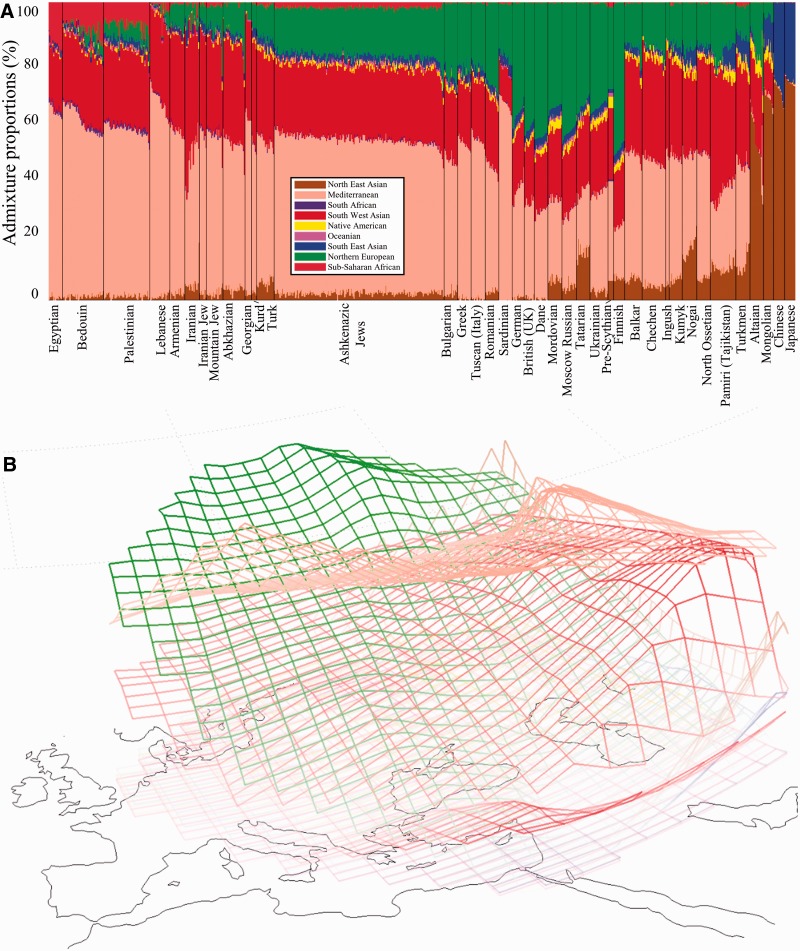
Depicting the distributions of nine admixture components. (*A*) Admixture proportions of all populations included in this study. For brevity, subpopulations were collapsed and only half of all AJs are presented (see supplementary fig. S3, Supplementary Material online, for the full distribution). The *x*-axis represents individuals. Each individual is represented by a vertical stacked column of color-coded admixture proportions that reflects genetic contributions from nine putative ancestral populations. (*B*) The geographical distribution of admixture proportions in Eurasia.

Prior to applying GPS to elucidate the geographical origins of AJs, we sought to evaluate its accuracy on Afro-Eurasian populations. For that, we analyzed the genomes of over 600 individuals belonging to 35 populations and estimated their admixture proportion in respect to nine admixture components corresponding to putative ancestral populations (fig. 2*A*). All the genomes consist of at least four admixture components and segregate within and among neighboring populations. In western Eurasians, Mediterranean, Southwest Asian, and Northern European are the most dominant admixture components with the latter nearly replacing the sub-Saharan component (fig. 2*B*). Genetic diversity was estimated by computing the genetic distances (*d*), defined as the minimal Euclidean distances between the admixture proportions of each individual and all members of a population of interest. Small genetic distances indicate high genetic similarity. The median genetic distances in all populations are small ($\bar{d}$= 2.13 ± 2.13%), suggesting high within-population homogeneity.

**Fig. 3.—:**
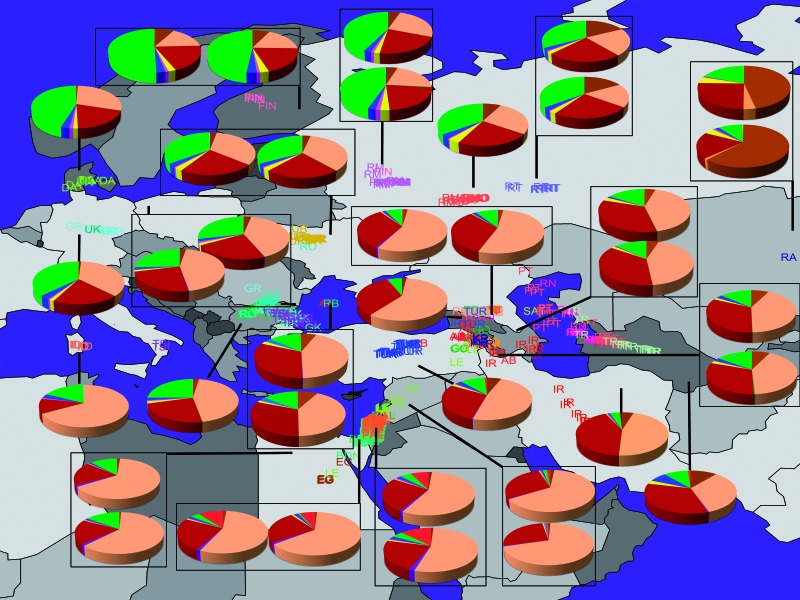
GPS predicted coordinates for individuals of Afro-Eurasian populations and subpopulations. Individual labels and colors match their known region/state/country of origin using the following legend: AB (Abkhazian), ARM (Armenian), BDN (Bedouin), BU (Bulgarian), DA (Dane), EG (Egyptian), FIN (Finnish), GK (Greek), GO (Georgian), GR (German), ID/TSI (Italy: Sardinian/Tuscan), IR (Iranian), KR (Kurds), LE (Lebanese), Palestinian (PAL), PT (Pamiri from Tajikistan), R-A/B/C/I/K/MO/N/NO/T (Russia: Altaian/Balkar/Chechen/Ingush/Kumyk/Mordovian/Nogai/North Ossetian/Tatar and RM for Moscow Russians), RO (Romanian), TR (Turkmen), TUR (Turk), UK (United Kingdom), UR (Ukranian). Pie charts reflect the admixture proportions and geographical locations of the reference populations. *Note*: occasionally all individuals of certain populations (e.g., Altaians) were predicted in the same spot and thus appear as a single individual.

We applied GPS using the leave-one-out procedure at the population level. Assignment accuracy was determined for each individual based on whether the predicted geographical coordinates were within 500 or 250 km from the political boundaries of the individual’s country or regional locations. GPS correctly assigned 83% and 78% of the individuals within <500 and 250 km from their countries, respectively (fig. 3 and supplementary table S2, Supplementary Material online). The low prediction accuracy for some populations (e.g., Chinese) can be explained by the low density of reference populations in their areas or high genetic heterogeneity (e.g., Altaians). Within the area covered by the two linguistic hypotheses and harbored by 554 individuals belonging to 31 populations, the accuracy was 2% higher. As expected, the prediction accuracy within that area was even higher (97% and 94% of the individuals were assigned within <500 and 250 km of their countries, respectively) for speakers of geographically localized languages (Abkhazians, Armenians, Bulgarians, Danes, Finns, Georgians, Greeks, Romanians, Germans, and Palestinians), which also include some of the putative basal components of Yiddish (Romance, Slavic, Hebrew, and German). These results illustrate the tight relationship between genome, geography, and language and delineate the expected assignment accuracy for Yiddish speakers.

### Biogeographical Mapping of Eurasian Jews

**Fig. 4.—:**
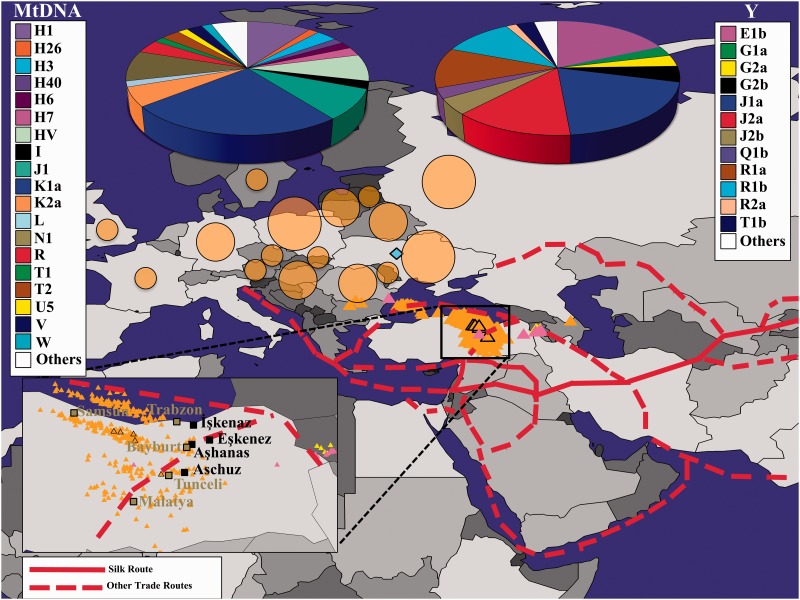
A map depicting the predicted location of Jewish (triangles) AJs (orange), claimants of priestly lineages (orange and black), Mountain Jews (pink), and Iranian Jews (yellow) alongside the ancient pre-Scythian individual (blue diamond). An inset shows the sample distribution in northern Turkey, the locations of the four villages that may derive their names from “Ashkenaz,” and adjacent cities. Large (13–23%), medium (4–10%), and small (1–4%) circles reflect the percentage of AJs’ parents born in each region. The paternal and maternal haplogroups of the AJs are shown at the top of the figure.

Like most Eurasians, Yiddish speaker genomes are a medley of three major components: Mediterranean ($\bar{X}$= 52%), Southwest Asian ($\bar{X}$= 24%), and Northern European ($\bar{X}$= 16%) (fig. 2*A*), although, like the ancient pre-Scythian, they also exhibit a small and consistent sub-Saharan African component ($\bar{X}$∼2%), in general agreement with Moorjani et al. (2011). GPS positioned nearly all Ashkenazic Jews (AJs) on the southern coast of the Black Sea in northeastern Turkey adjacent to the southern border of ancient Khazaria ($\tilde{40{}^{\circ}41’}$N, $\tilde{37{}^{\circ}39’}$E) (fig. 4). There we located four primeval villages that bear names that may derive from “Ashkenaz”—İşkenaz (or Eşkenaz) at (40°9′N, 40°26′E) in the province of Trabzon (or Trebizond), Eşkenez (or Eşkens) at (40°4′N, 40°8′E) in the province of Erzurum, Aşhanas (today Üzengili) at (40°5′, 40°4′E) in the province of Bayburt, and Aschuz (or Hassis/Haza, 30 B.C.–A.D. 640) (Bryer and Winfield 1985; Roaf et al. 2015) in the province of Tunceli—all of which are in close proximity to major trade routes. The Turkish toponyms/ethnonyms are very suggestive of a Jewish trading presence, but given the poor state of Turkish toponymic studies, we cannot say for sure. There are no other place names anywhere in the world derived from this ethnonym. Instead, to the best of our knowledge, the many Jewish “way stations” on the trade routes throughout Afro-Eurasia are named after the root “Jew” (Wenninger 1985), but these may be places named by non-Jews. AJs were localized within $\tilde{211}km$ from at least one such village. Similar results were obtained with Turks excluded from the reference panel indicating the robustness of our approach (results not shown). No individual was positioned in Germany or proximate to the ancient pre-Scythian individual who was localized to Ukraine, ∼500 km from Ludas-Varjú-Dűlő in Hungary where it was originally found. A comparison of the genetic distances between AJs and the reference populations (supplementary fig. S2, Supplementary Material online) confirmed that AJs are significantly closer to Turks ($\tilde{d}$ = 9.2%), Armenians ($\tilde{d}$ = 11.5%), and Romanians ($\tilde{d}$ = 12.28) than to other populations (Kolmogorov–Smirnov goodness-of-fit test, *P *<**0.01). The genetic distance to Germans ($\tilde{d}$= 26.81%) was slightly higher than to the pre-Scythian individual ($\tilde{d}$= 22.4%).

Similar results were found for other Jewish communities and AJ subgroups. Iranian Jews were positioned ∼200 km east of Eşkenez close to Tabriz where a large Jewish community existed during the first millennium (Gilbert 1993). The Mountain Jews nested with and between both Jewish communities forming a geo-genetic continuum. The admixture and GPS results for Yiddish and non-Yiddish speakers were very similar. On average, these two cohorts have the same admixture components (supplementary fig. S3, Supplementary Material online), and their geographical origins follow similar trends (supplementary fig. S4, S5 Supplementary Material online). That all AJs were predicted away from their parental birth countries (fig. 4) implies arrival by migration and limited gene exchange with Western and Central European populations.

### Haplogroup Analysis of AJs

For AJs, the most common (frequency ≥5%) low-resolution mtDNA haplogroups explain less of the variation compared to the Y haplogroups. More specifically, the most common mtDNA haplogroups K1a, H1, N1, J1, HV, and K2a are present in 65% of the individuals compared with 74% of the individuals that belong to the most common Y haplogroups J1a, E1b, J2a, R1a and R1b. The top six most common high-resolution mtDNA (K1a1b1a [16.89%], N1 [7.36%], K1a9 [6.54%], K2a2a [4.36%], HV1b2, and HV5 [3.54% each]) and Y (R1a1a2a2 [8.98%], J1a1a1a1a1 [7.76%], E1b1b1b2a1a [6.93%], J1a1a1 [5.31%], R1b1a1a [4.9%], and G2b1 [4.49%]) haplogroups are present in about a third of the samples. We observed major dissimilarities in the number of unique Y chromosomal and mtDNA haplogroups between Yiddish (46 and 69, respectively) and non-Yiddish speakers (46 and 63, respectively) who exhibit lower haplogroup diversity (supplementary figs. S4 and S5, Supplementary Material online). Yiddish speakers belong to maternal lineages like H7, I, T2, and V alongside the paternal Q1b—all are rare or absent in non-Yiddish speakers (supplementary table S3, Supplementary Material online). Nearly all common high-resolution haplogroups appear more frequently in Jews than non-Jews, though none are unique to AJs or Jews in general and three of them are infrequent in AJs compared with other groups (supplementary fig. S6, Supplementary Material online).

The most common Y haplogroups dominate the area between the Black and Caspian Seas and represent the major lineages among populations inhabiting Western Asian regions, including Turkey, Iran, Afghanistan, and the Caucasus (Yardumian and Schurr 2011; Cristofaro et al. 2013; Tarkhnishvili et al. 2014). In contrast, the mtDNA haplogroups indicate a more diffused origin and include haplogroups common in Africa (e.g., L2), Near East (e.g., J), Europe (e.g., H), North Eurasia (e.g., T and U), Northwest Eurasia (e.g., V), Northwest Asia (e.g., G), and Northeast Eurasia (e.g., X) (Jobling et al. 2013). High-genetic diversity was also observed in the Y (I2, J1a1a1a1a1, R1a1a2a2) and mtDNA haplogroups (K1a1b1a, N1, HV1b2, K1a, J1c5) of priestly lineage claimants.

### The Geographical and Ancestral Origins of AJs

GPS findings raise two concerns: first that the Turkish “Ashkenaz” region may be the centric location of other regions rather than the place where the Ashkenazic Jewish admixture signature was formed; second, in the absence of “Ashkenazic” Turks it is impossible to compare the genetic similarity between the two populations to validate the common origins implied by the GPS results.

To surmount these problems we derived the admixture signatures of “native” populations corresponding to the geographic coordinates of interest from the global distributions of admixture components (fig. 2*B*) and compared their genetic distances with AJs. This approach has several advantages. First, it allows studying “native” populations that were not sampled. Second, it allows identifying putative progenitors by comparing genetic distances between different populations. Third, it minimizes the effect of outliers in modern-day populations. Finally, it circumvents, to a certain degree, the problem of comparing AJs with modern-day populations that may have experienced various levels of gene exchange or genetic drift past their mixture with AJs.

**Fig. 5.—:**
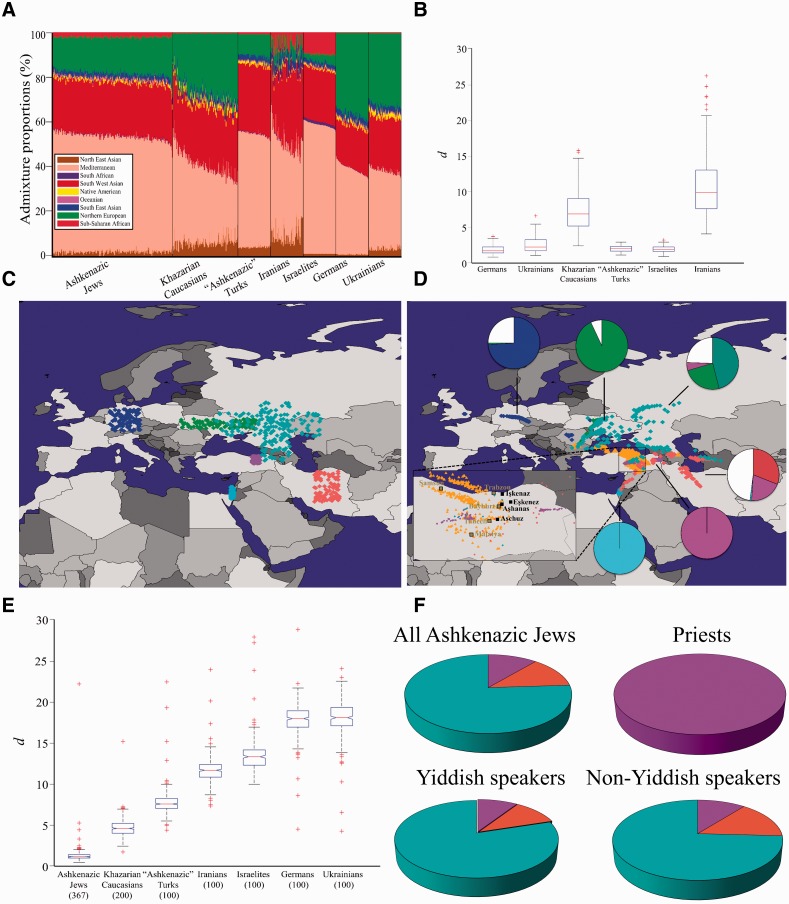
Comparing AJs with “native” individuals from six populations. (*A*) Admixture proportions of AJs and all simulated individuals included in this analysis. For brevity, only half of all AJs are presented. The *x*-axis represents individuals. Each individual is represented by a vertical stacked column of color-coded admixture proportions that reflects genetic contributions from nine putative ancestral populations. (*B*) The genetic distances (*d*) between the simulated individuals and their nearest modern-day populations. (*C*) The geographical coordinates from which the admixture signatures (*A*) were derived. (*D*) GPS predictions for the admixture signatures of the simulated individuals of the six populations. Pie charts denote the proportion of individuals correctly predicted in the countries of origins, coded by the colors of the six countries (*C*) or white for other countries. The geographical origins of Yiddish speakers previously obtained are shown for comparison. An inset magnifies northeastern Turkey. (*E*) The *d* within Yiddish speakers and between them to the simulated individuals. (*F*) The proportion of simulated individuals that are geographically closest to Ashkenazic Jewish subgroups.

We generated the admixture signatures of 100 or 200 “native” individuals from six areas associated with the origin of Yiddish and AJs (fig. 4, supplementary figures S4 and S5, Supplementary Material online, and table 1): Germany, Ukraine, Khazaria, Turkish “Ashkenaz,” Israel, and Iran (fig. 5A and *C*). We first tested the genetic affinity of these “native” populations by examining their genetic distances (*d*) to modern-day populations residing within the same regions (fig. 5*B*). For Israelites, we used Palestinians and Bedouins, and for Khazars we used Armenians, Georgians, Abkhazians, Chechens, and Ukrainians. The average $\tilde{d}$ between the native and modern-day populations was 4, slightly higher than within modern-day populations (supplementary fig. S1, Supplementary Material online), with Khazarian and Iranian showing the highest heterogeneity. Consequently, GPS mapped most of the “native” individuals to their correct geographical origins (fig. 5*D*), with the exception of the Khazars and Iranians, likely due to the shared historical, geographical, and genetic backgrounds of Iranians, Turks, and southern Caucasus populations (Shapira 1999).

The AJs predicted in our earlier analysis (fig. 4) largely overlapped with “native” “Ashkenazic” Turk and a few Khazarian and Iranian individuals mapped to northeastern Turkey. A comparison of *d* between the AJs and “native” populations (fig. 5*E*) confirmed that Yiddish speakers are significantly (Kolmogorov–Smirnov goodness-of-fit test, *P *<**0.01) closer to each other ($\tilde{d}$= 1.1%), followed by “native” Khazars ($\tilde{d}$= 4.6%), “Ashkenazic” Turks ($\tilde{d}$= 7.7%), Iranians ($\tilde{d}$= 11.9%), Israelites ($\tilde{d}$= 13.6%), Germans ($\tilde{d}$= 18.3%), and Ukrainians ($\tilde{d}$= 18.5%). Similar results were obtained for Yiddish and non-Yiddish speakers (supplementary figs. S7 and S8, Supplementary Material online). Whereas most AJs are geographically closest to “native” Khazars (76%), followed by Iranian (13%) and *“Ashkenazic” Turks (11%), priestly lineage claimants are closest to “native” “Ashkenazic” Turks* (fig. 5F).

**Fig. 6.—:**
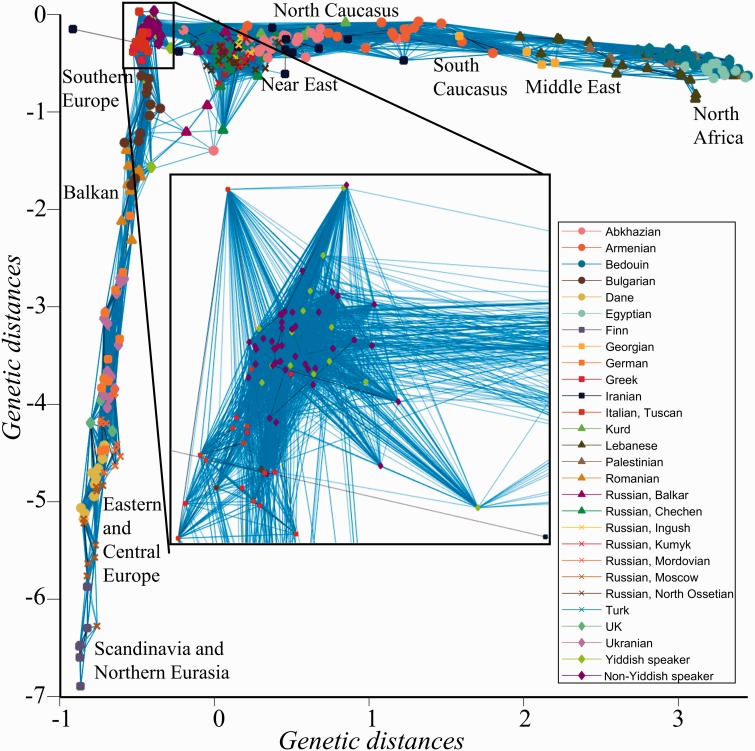
Undirected graph illustrating the genetic distances (*d*) between all non-Jewish individuals included in this study. An inset shows the distances between AJs (Yiddish and non-Yiddish speakers) and populations with whom they share small *d*. For coherency, edges are shown between genetically similar individuals (*d *<**0.75). Some Iranians, Sardinians, Tajiks, Altai, and East Asians clustered separately and are not shown.

To identify additional potential founding populations, we assessed the genetic distances between AJs and all non-Jewish individuals in this study, including populations excluded from the reference population panel. Most of the individuals cluster along an ‘A’-shaped structure with the ends corresponding to Scandinavians and North Africans. AJs, due to their large number, formed the apex of the ‘A’, connecting Southern Europeans with Near Eastern (fig. 6). AJs overlapped with few Greeks and Italians within an Irano-Turkish super-cluster.

The relative dearth of individuals related to both AJs and Near Eastern populations can be explained in several ways. First, key founding populations are either missing from our study, are highly heterogeneous and underrepresented in our study (e.g., Iranians), or have disappeared over time through demographic processes. This hypothesis can be addressed in future studies with additional samples from this region. Second, the loss of millions of Eastern and Western European Jews during the mid-20th century may account for the observed gap. Though this hypothesis cannot be formally tested, we note that six AJs of German descent cluster at the center of the AJs distribution or north of it, whereas six other AJs positioned at the south and east edges of that distribution were of Eastern European descent. Third, Ashkenazic Jewish genomes may be conglomerates of Greco-Roman-Turko-Irano-Slavic and perhaps Judaean genomes (Wexler 1993; Sand 2009; Moorjani et al. 2011; Elhaik 2013) formed through ongoing proselytization events that continued undisturbed for many centuries in Turkish “Ashkenaz.” These events were localized to the extent that no single Ashkenazic non-Jewish population presently exists. However, the few Greek, Italian, Bulgarians, and Iranian individuals clustered with or adjacent to AJs imply that individuals descent from the potential progenitors of AJs still exhibit similar genetic makeup to AJs and may even be at risk for the genetic disorders prevalent in this population (Ostrer 2001). Confirming this hypothesis will shed new light on the origin of mutations associated with genetic disorders, like Cystic fibrosis (OMIM #219700) and α-thalassaemia (OMIM #141800) and promote genetic screening for all at risk individuals. Identifying the founding populations and their relative contribution to the AJ genome necessitate using biogeographical tools that can discern multiple origins, but such an analysis is beyond the scope of this article.

## Discussion

Every language is the creative product of a community and a co-creator of behavior and values, but Yiddish has experienced especially extreme peregrinations as the millennia-old vernacular of AJs. The questions of Yiddish and AJ origins have been some of the most debatable questions in history, linguistics, and genetics over the past 300 years. While Yiddish is clearly a blend of at least three languages—German, Slavic, and Hebrew—the exact proportions, and consequently its geographical origin, remain unsettled (table 1, fig. 1). Weinreich (2008) emphasized the truism that the history of Yiddish mirrors the history of its speakers, which prompted us to reconstruct the geographical and ancestral origins of Yiddish and non-Yiddish speaking AJ genomes. These analyses revealed the birthplaces of Yiddish and AJs.

### Evaluating the Evidence for the Geographical Origin of AJs

Regardless of linguistic orientation, descendants of Ashkenazic Jewish parents comprised mostly a homogeneous group in terms of genetic admixture and geographic origins. Intriguingly, GPS positioned nearly all AJs in the vicinity of the ancient Scythian-inhabited territory, in close proximity to four primeval villages: İşkenaz, Eşkenez, Aşhanas, and Aschuz that may derive their names from “Ashkenaz” (fig. 4). Historically, the area where these villages were found was in the Greek Kingdom of Pontus (Bryer and Winfield 1985) established by Greek settlers in the early first millennium who took active part in maritime trade (Drews 1976). Prior and sporadically through the early 10th century, that area was a center of Byzantine commercial and coastal trade, inhabited by a Jewish community (Holo 2009). We surmise that the admixture signature of Ashkenazic Jewish genomes was formed in this major transcontinental hub connecting East Asian, West European, and North Eurasian roads. Most of the AJs were localized between Trabzon and Amisus (today Samsun), found ∼300 km west of Trabzon, where a widespread Jewish settlement existed during the early centuries A.D. Primeval Iraqi Jewish communities proliferated by 600 A.D., like Sarari, Nisibis (today Nusaybin), and Argiza could be found ∼300 km south to the Bayburt province (Gilbert 1993).

Remarkably, our findings echo Harkavy’s, who wrote in 1867 that “the first Jews who came to the southern regions of Russia did not originate in Ashkenaz [Germany], as many writers tend to believe, but from the Greek cities on the shores of the Black Sea and from Asia via the mountains of the Caucasus” (Harkavy 1867), and those of anthropologist Weissenberg (Efron 1994). Our findings also support Rabinowitz’s thesis that European Jewish communities often nested along continental trade routes, which determined their preferred residency. Rabinowitz argued in favor of “an unbroken chain of Jewish communities” from the West to the Far East upon which Jews, and particularly the Radhanites, could rely for their travels (Rabinowitz 1948).

Thus, far only few studies attempted to trace the geographical origins of AJs. Our results are in general agreement with two small-scale studies: the first positioned 20 Eastern (38 ± 2.7°N, 39.9 ± 0.4°E) and Central (35 ± 5°N, 39.7 ± 1.1°E) European Jews south of the Black Sea (Elhaik 2013), ∼100 km away from the province of Tunceli. The second reported an Eastern Turkish origin (41°N, 30°E) for 29 AJs (Behar et al. 2013), ∼630 km west of the mean geographical coordinates obtained here.

### Evaluating the Evidence for the Ancestral Origins of AJs

Although our biogeographical results are well localized, the exact identity of AJ progenitors remains nebulous. The term “Ashkenaz” is already a tantalizing clue to the large Iranian-origin group that inhabited the central Eurasian steppes, though it cannot be considered evidence of a Scythian origin due to the lack of records about Scythian culture and the obsolescence of Scythian language about 500 years prior to the appearance of Yiddish. It is more likely that AJs called themselves “Scythians” because this was a popular name in the Bible and in the Caucasus–Ukraine area even long after the disappearance of the Scythians. AJs may have even considered themselves related to the Scythians based on a shared Irano-Turkish origin, as evident from the proximity of Yiddish speakers to Iranian Jews, positioned close to Iran; however, they probably were not Scythians. Irano-Turkish Jews were speakers of Persian, Ossete, or other forms of Iranian, which became extinct during the 10th century. This conclusion is further corroborated by the large geographical distance between the predicted origins of AJs and the ancient pre-Scythian (fig. 4).

The inheritance patterns of the mtDNA chromosomes are directly related to the question of Ashkenazic Jewish origins. Costa et al. (2013) reported that four major founding mtDNA lineages account for ∼40% of mtDNA variation in AJs (K1a1b1a [20%], K1a9 [6%], K2a2a1 [5%], and N1b2 (N1b1b) [9%]). These haplogroups were among the six most common haplogroups in our analyses and accounted for 37.6% and 39.5% of the mtDNA variation among Yiddish and non-Yiddish speakers, respectively. Costa et al. reasoned that Judaized women made major contributions to the formation of Ashkenazic communities. This conclusion is in agreement with a widespread Judaization of slaves (Sand 2009) and depictions of Greco-Roman women leading communities of proselytes and adherents to Judaism during the first millennium, A.D. (Kraemer 2010).

Another clue to the diverse background of AJs’ progenitors is the limited haplogroup diversity among non-Yiddish speakers that may indicate the loss of rare haplogroups, probably through genetic drift since they are uncommon in Europe. For example, the Northern Asiatic Q1b1a Y haplogroup, one of the most common haplogroups among Yiddish speakers (3.7%), is completely absent among non-Yiddish speakers. Far Eastern maternal haplogroups found in AJs were recently reported by Tian et al. (2015). The mitochondrial haplogroup L2a1 is found in five Ashkenazic maternal lineages, where 80% of the mothers speak solely Yiddish (supplementary table S3, Supplementary Material online). A search in the Genographic public dataset found 229 individuals with that haplogroup. Of those, 169 described their maternal descent as African (156), European (4), or “Jewish” (9), mostly Ashkenazic.

One of the most fascinating questions in genetics is the origin of individuals whose surnames hint of an association with Biblical priesthood lineages. The haplogroup diversity of the five priestly lineage claimants, positioned close to simulated “Ashkenazic” Turks (fig. 5*F*), suggests that they have originated from shamans who adopted the surname, in support of historical descriptions of Jews establishing a proselytization center in “Ashkenaz” lands where they have anointed Levites and Cohens to Judaize their slaves and neighboring populations (Baron 1937). Interestingly, Brook (2014) reported a Crimean Karaite man with a surname of *Kogen* who self-identifies as a Cohen and belongs to a J1 (J-M267) Y haplogroup. His panel of 12 short-tandem repeats (STRs) on that chromosomal, but not a panel of 25 STRs, matched exactly a Belarusian Ashkenazic Cohen whose surname is *Kagan* (*Kahan*). We surmis that some Cohen surnames are later modifications of *Kagan* (*Kahan*), the term used by Turks and Khazars to denote a leader. This hypothesis may explain the difficulties in establishing genetic markers associated with priesthood (Zoossmann-Diskin 2006; Klyosov 2009; Tofanelli et al. 2009, 2014) despite the assiduous and indefatigable efforts to do so (e.g., Skorecki et al. 1997; Thomas et al. 1998; Nebel et al. 2000, 2001; Behar et al. 2003; Hammer et al. 2009; Rootsi et al. 2013). In the era of ancient DNA sequencing, the peculiar absence of priestly or even Judaean ancient DNA should render any assertions or insinuations that certain genetic markers are telltales of Judaean lineages or Biblical figures as fictitious.

Our autosomal analyses highlight the high genetic similarity between AJs and Iranians, Turks, southern Caucasians, Greeks, Italians, and Slavs (figs. 6 and 4*D*, and supplementary fig. S1, Supplementary Material online). Altogether, our results portray a millennium-old melting-pot process in the focal region of Turkish “Ashkenaz” that crystallized these and other putative progenitors into an Ashkenazic Jewish community in agreement with the first prediction of the Irano-Turko-Slavic hypothesis (table 1, fig. 1). Our findings further imply that the migration of AJs to Europe was followed by social isolation and avoidance of intermarriages, which largely retained their unique admixture signature, although we cannot rule out the possibility of a limited gene exchange and religious conversions. Nonetheless, socioreligious practices compounded with a unique language seems to be more effective means of genetic isolation than geographical barriers (Elhaik 2012).

Our findings are also consistent with the vast majority of genetic findings that AJs are closer to Near Eastern (e.g., Turks, Iranians, and Kurds) and South European populations (e.g., Greeks and Italians) as opposed to Middle Eastern populations (e.g., Bedouins and Palestinians). Remarkably, with only few exceptions (e.g., Need et al. 2009; Zoossmann-Diskin 2010), these findings have been consistently misinterpreted in favor of a Middle Eastern Judaean ancestry, although the data do not support such contention for either Y chromosomal (Hammer et al. 2000; Nebel et al. 2001; Rootsi et al. 2013) or genome-wide studies (Seldin et al. 2006; Kopelman et al. 2009; Tian et al. 2009; Atzmon et al. 2010; Behar et al. 2010; Campbell et al. 2012; Ostrer and Skorecki 2012). To promulgate a Middle Eastern origin despite the findings, various dispositions were adopted. Some authors consolidated the Middle East with other regions whereas other authors abolished it altogether. For example, Seldin et al. (2006) wrote that the “southern [European]” component is “consistent with a later Mediterranean origin,” whereas Rootsi et al. (2013) declared it as part of the Near East, which is “the geographic location for the ancient Hebrews” and, apparently, Ashkenazic Levites. A common fallacy is interpreting the genetic similarity between AJs as evidence of a Middle Eastern origin. For example, Kopelman et al. (2009) advised caution when considering the similarity between AJs with Adygei and Sardinians and since Jewish communities clustered together they “share a common Middle Eastern ancestry.” Tian et al. (2009) dismissed similar findings for AJs, denouncing them as the only population that “appears to have a unique genotypic pattern that may not reflect geographic origins.” A newly emerging trend is partial “Middle Easternization.” For example, Behar et al. (2013) traced AJs to eastern Turkey but argued in favor of a shared Middle Eastern and European ancestries based on the shared ancient Middle Eastern origin, common to most Near Eastern populations. This approach assumes undisturbed genetic continuity of AJs since the Neolithic Era along with the existence of a Middle Eastern ancestral component—both are unsupported by the data. In fact, all western and central Eurasians share similar admixture components (fig. 2*A*) and “Middle Easternalizing” is uninformative to study recent origin, particularly when applied selectively to populations who exhibit similarity to AJs. Similarly, Atzmon et al. (2010) have reported that Northern Italians show the greatest proximity to AJs, followed by Sardinians and French, in support of non-Semitic Mediterranean ancestry, but the coloring patterns of their admixture plot (which are similar to our fig. 2*A*) persuaded them that AJs have “demonstrated [a] Middle Eastern ancestry.” Most innovatively, the authors have then interpreted the differential patterns of genetic segments that are identical-by-descent (IBD) in AJs as consistent with a bottleneck paradigm citing a “demographic miracle” to support this claim. To the best of our knowledge, no large-scale study has reported that AJs are genetically closer to German or Israelite populations compared with Near Eastern and Southern European populations. Bedouins and Palestinians are the only populations localized to Israel (fig. 3).

### Evaluating the Evidence for the Rhineland Hypothesis

The Rhineland hypothesis is unsupported by our analyses and suffers from several weaknesses. First, it relies on an unsubstantiated event purported to explain how Judaeans arrived in Eastern Europe from Judea or Roman Palestine (Sand 2009). Second, it consists of major migrations from Germany to Poland that did not take place (van Straten 2003). Third, it dismisses the contribution of proselytes by assuming a “demographic miracle” that inflated only the Jewish population size in Eastern Europe from 50,000 (15th century) to 5 million (19th century) (Ben-Sasson 1976; Atzmon et al. 2010; Ostrer 2012), already criticized by several authors (e.g., van Straten and Snel 2006; Elhaik 2013). Ironically, mysticism, superstitions, and other supernatural elements have likely been introduced to AJs by Judaized pagans (Wexler 1993; Efron 1994). Fourth, it ignores the small size of the Jewish population in Middle Ages Germany that was on the order of hundreds or thousands, which makes them unlikely to exact a strong cultural influence on the numerous Irano-Turko-Slavic AJs (Polak 1951) or meaningful genetic contribution as is evident by the Irano-Turko-Slavic admixture signature of AJs (figs. 4–6). This genetic contribution has already been reported in epidemiological studies. For example, studying rare skin disorders Mobini et al. (1997) reported that AJs and northwest Iranian non-Jews carry the same major histocompatibility complex haplotypes for Pemphigus Vulgaris. The authors surmised that this gene arose before the separation of the two populations. Crucially, much of the “German” component that buttresses the Rhineland hypothesis are actually “Germanoid” elements that deviate from native German norms and were invented by Yiddish speakers, mainly based on Slavic and, to a lesser extent, on Iranian models (Wexler 1999, 2012). It is also unclear why Semitic Hebrew, which had been dead for nearly a millennium, would be revived in the 9th century.

Some of the confusion contributing to the establishment of this hypothesis stems from the erroneous association of the term “Ashkenaz” with “German lands, Germans (Jews and non-Jews)” in the late 11th century, contemporaneous with the rise of Yiddish (Wexler 2011b). Ashkenazic began with the meaning of “Scythian.” In the 10th century in Baghdad it meant “Slavic” and by the early 1100s in Europe it assumes the meaning of German/Yiddish, and later the German non-Jews and the German lands. In the 10th century, a Moroccan Karaite philologist knew that the Ashkenazic people descended from Khazars and “Germans”—meaning that they came from the Khazar Empire and spoke Yiddish. The author of a Hebrew–Persian dictionary from Urgench (present-day Uzbekistan) in the early 14th century called his native land “Ashkenaz.” In the early 20th century, Caucasian Jews were still known by their Lezgian neighbors as “Ashkenazic” (Byhan 1926). The surname Ashkenazic was also occasionally found among the Crimean Krimchaks (Weinreich 2008).

### Reconstructing the Origin of AJs and Yiddish

The most parsimonious explanation for our findings is that Yiddish speaking AJs have originated from Greco-Roman and mixed Irano-Turko-Slavic populations who espoused Judaism in a variety of venues throughout the first millennium A.D. in “Ashkenaz” lands centered between the Black and Caspian Seas (figs. 4 and 5) (Baron 1937). These pagans became Godfearers (non-Jewish supporters of Second Temple Judaism) probably around the first century A.D. after encountering Irano-Turkish Jews and have accepted the doctrine of Judaism to the extent that they created at least two translations of the Bible into Greek during the first and second centuries. They were also experienced maritime merchants who may have considered the mutual advantages in forming an alliance with the Irano-Turkish Jews.

At the height of the Khazar Empire (8th–9th centuries), Hebrew as a native language had been dead for five to six centuries. In the Empire, Slavic and Iranian had become major *lingua francas* (Wexler 2010). At this time, Iranian Jews had brought to the Khazar Empire an Iranianized Judaism, together with the Talmud, as well as written Talmudic Aramaic, Biblical Hebrew, written Hebroid, and spoken Eastern Aramaic and Iranian. The Khazars converted to Judaism to profit from the transit trade across their territories. They appear not to have participated very much as merchants abroad. The Judaization of the Khazar élite and the presence of the international Jewish merchants plying the international Silk Roads between China, the Islamic world, and Europe (Baron 1957; Noonan 1999) prompted the Irano-Turko-Slavo Jewish merchants to create Yiddish for use in Europe, Loterā’i (a cryptic language first cited in 10th century Azerbaijan and surviving to the present day) for use in Iran, and the many variants of cryptic Hebrew and Hebroid lexicon for the use of Jewish merchants throughout Afro-Eurasia (Wexler 2010). This is evident in both genetic and linguistic evidence: by the biogeographical proximity of Yiddish speakers to Iranian, Iranian Jews, and Turks (figs. 4–6) and the existence of over 250 terms meaning “buying and selling” in Yiddish, most of which were Hebroidisms, Germanoidisms, and Slavisms, with only a handful of authentic German terms (Wexler 2011a). The existence of Jewish communities along major trade routes (Rabinowitz 1945) who share religion, common Irano-Turko-Slavic culture, and history (figs. 4 and 5), and a secret language (Wexler 1993) created a political and spiritual unity and maintained a Jewish trading advantage. We note that while Hebrew could serve as the basis of the international cryptic trade lexicon, it could not serve as a full-fledged language since no Jew could speak the language by that time.

In the 9th century, a Persian postal official in the Baghdad Caliphate, ibn Khordādhbeh, described the Iranian Jewish traders, who by then may have already become a tribal confederation of Slavic, Iranian, and Turkic converts to Judaism, as conversant in the main components of Yiddish: Slavic, German, Iranian, Hebrew, in addition to several other languages. The total number of languages given was six, but some of his language names were most likely abbreviations of sets of languages, for example, *’andalusijja*’ probably denoted Andalusian Arabic, Berber, and various forms of Ibero-Romance.

When the Khazar Empire lost its prominence and the Jewish monopoly on the Silk Road ended (∼11th century), the relexification process was gradually abandoned (Wexler 2002). At that point, Slavic Yiddish became the first and only spoken and written language of the European AJs (Iranian remained the language of the Central Asian and Iranian AJs—and both groups continued to call themselves “Ashkenazic” up to the present) and began to absorb more German influence post-relexificationally (Wexler 2011a). Consequently, Yiddish grammar and phonology are Slavic (with some Irano-Turkic input) and only some of the lexicon is German (Wexler 2012). This process, however, was not accompanied by massive gene exchanges between Jews and non-Jews (fig. 4), likely due to the severe restrictions set on mixed marriages by the Medieval Christian authorities (Sand 2009). This is also consistent with the estimated dates of admixture in AJ genomes (695–1,215 A.D.) (Moorjani et al. 2011). If one examines the “German” and “Hebrew” component of contemporary Yiddish, one can still see the enormity of the Germanoid and Hebroid components in comparison to genuine Germanisms and Hebraisms. To take one example, Yiddish *unterkojfn* ‘to bribe’ has German components (‘under’ + ‘to buy’), but the combination and meaning are impossible in all forms of German, past or present (Wexler 1991).

Further evidence to the origin of AJs can be found in the many customs and their names concerning the Jewish religion, which were probably introduced by Slavic converts to Judaism. For example, the Yiddish term *trejbern* ‘to remove the forbidden parts of the animal to render the meat kosher’ is from Slavic, for example, Ukrainian *terebyty* means ‘to peel, shell; clean a field’ (the Yiddish meaning is obviously innovative). Another Ashkenazic custom of distinctly non-Jewish is the breaking of a glass at a wedding ceremony (Slavic and Iranian) (Wexler 1993). A striking fact that is hardly ever appreciated is that Yiddish *košer* ‘kosher’ is not a Hebraism, as is widely believed (it appears centuries after the demise of colloquial Semitic Hebrew), but the source of the term is a common Iranian word meaning ‘to slaughter an animal’, for example, Ossete *kušart* means ‘animal slaughtered for food.’ Apparently, Yiddish speakers “Hebroidized” the Iranianism with the legitimate Biblical Hebrew *kašer* which meant only ‘fit, suitable’ but had no connection to food. Many of the Arabic-speaking Jews to this day do not use the Hebrew/Hebroid term at all.

Our findings illuminate the historical processes that stimulated the relexification of Yiddish, one of over two dozen other languages that went through relexification, like Esperanto (Yiddish relexified to Latinoid lexicon), some forms of contemporary Sorbian (German relexified to Sorbian lexicon) and Ukrainian and Belarusian (Russian relexified to Ukrainian and Belarusian lexicon) (Horvath and Wexler 1997).

### Limitations

Our study has several limitations. First, because our study is the first to analyze the genomes of Yiddish speaking AJs, a caution is warranted in interpreting some of our results due to the choice of data, method, and individuals. Second, DNA samples were genotyped on the GenoChip (Elhaik et al. 2013), which is relatively small in size and does not allow extensive IBD analyses, although previous IBD findings agree with our findings (Elhaik 2013). Third, using contemporary populations may have restricted our ability to identify all the historical progenitors of AJs. Fourth, since our biogeographical approach requires using homogeneous cohorts, the genetic makeup of AJs, reported here, represents only a segment of the genetic diversity of this community. A search in the Genographic dataset indicates that the broader Ashkenazic Jewish community, which consists of mixed couples of non-Ashkenazic or non-Jewish origins, is twice the size of the cohort we studied and likely more genetically heterogeneous. Finally, GPS infers the geographical origins of an individual by averaging over the origins of all its ancestors, raising doubts as to whether the reported area is the actual origin or middle point of several origins. We have accounted for that by carrying out a separate analysis that confirmed the high genetic similarity between AJs, modern Turks (supplementary fig. S2, Supplementary Material online), and simulated “native” “Ashkenazic” Turks (fig. 5).

## Conclusions

Language is the atom of a community, the molecule that binds its history, culture, behavior, and identity, and the compound that unites its geography and genetics. It is thereby not surprising that the origin of AJs remains the most enigmatic and underexplored topics in history. Since the linguistic approaches utilized to answer this question have thus far provided inconclusive results, we analyzed the genomes of Yiddish and non-Yiddish speaking AJs in search for their geographical origins. We traced nearly all AJs to major primeval trade routes in northeastern Turkey adjacent to primeval villages, whose names may be derived from “Ashkenaz.” We conclude that AJs probably originated during the first millennium when Iranian Jews Judaized Greco-Roman, Turk, Iranian, southern Caucasus, and Slavic populations inhabiting the lands of Ashkenaz in Turkey. Our findings imply that Yiddish was created by Slavo-Iranian Jewish merchants plying the Silk Roads between Germany, North Africa, and China.

## Methods

### Sample collection

#### Genetic Data of AJs

The National Geographic Society’s Genographic Project contains genetic and demographic data from over 320,000 anonymous participants (https://genographic.nationalgeographic.com/ last accessed 15/3/2016). Participants were genotyped on the GenoChip microarray that includes nearly 150,000 non-functional (Graur et al. 2013) highly informative Y-chromosomal, mitochondrial, autosomal, and X-chromosomal markers (Elhaik et al. 2013). All participants provided written informed consent for the use of their DNA in genetic studies. Jews represent ∼4% of individuals in the database, of which 55% have self-identified as AJs and 5% as Sephardic Jews.

Genetic and demographic data for public participants of the Genographic Project are available from the National Geographic Society pursuant to signing a license. Our search in this database (January 2015) for individuals of Ashkenazic Jewish descent retrieved 367 individuals who reported having two Ashkenazic Jewish parents. Demographic and genetic data (supplementary table S3, Supplementary Material online) were stripped from information that could lead to identification. The mtDNA notation corresponds to build B16 and the Y haplogroup notation corresponds to the 2015 tree. The mutations associated with the mtDNA and Y chromosomal haplogroups (2015 tree and B16 build, respectively) are listed in supplementary tables S4 and S5, Supplementary Material online, respectively. Haplogroup assignment was done bythe Genographic Project. Plink (1.07) was used to testthe relatedness among Yiddish speakers using the genome flag. The average PiHat was 1.8% and maximum PiHat was 5.14%, indicating the absence of close relatives in our data.

#### Genetic Data of an Ancient Pre-Scythian Individual

Raw reads for the ancient pre-Scythian Iron Age individual were generated by Gamba et al. (2014). Reads were processed through our standardized variant calling pipeline (Pirooznia et al. 2014). In brief, reads were aligned to the human reference assembly (UCSC hg19—http://genome.ucsc.edu/), allowing two mismatches in the 30-base seed. Alignments were then imported to binary bam format sorted and indexed. Optical duplicates were removed. High-quality alignments with a minimum mapping quality score of 20 were selected. The Genome Analysis Toolkit (GATK) (McKenna et al. 2010) (2.6) was used by employing a likelihood model to generate both SNP and small indel calls for the data using the GATK Unified Genotyper function. Variants were filtered for a minimum confidence score of 30 and minimum mapping quality of 20. An additional variant recalibration step was conducted and filters were applied for base quality score, strand bias, mapping quality rank sum, read position rank sum, and homopolymer stretches. SNP clusters (>3 SNPs per 10 bp window) were excluded. Finally, calls were converted to plink format. Overall, we obtained over 388,000 high confidence SNPs, of which we analyzed over 58,000 that overlapped with the GenoChip microarray.

#### Genetic Data of Reference Populations

To curate the reference population dataset and demonstrate the validity of our approach, we studied 602 unrelated individuals representing 35 populations and subpopulations with ∼16 samples per population (supplementary table S1, Supplementary Material online). About 250 individuals from 19 populations and subpopulations were obtained from the Genographic Project and the 1000 Genomes Project that were genotyped on the GenoChip microarray (Elhaik et al. 2014). Bedouins and Turks were obtained from Behar et al. (2010) and Palestinians were obtained from the HGDP dataset (Conrad et al. 2006). The remaining individuals were selected from 13 Eurasian populations for which localized geographical origin and sufficient data (>4 samples) were available (Yunusbayev et al. 2011). Eight Iranian Jews were obtained from Behar et al. (2013) and 18 Mountain Jews were obtained from Karafet et al. (2015). From all these datasets, we analyzed only the ∼100,000 autosomal markers that overlapped with the GenoChip markers. In the smaller Karafet et al. (2015) dataset, ∼40,000 markers were analyzed.

### Curating a Reference Population Dataset

Biogeographical analysis was carried out using the GPS tool, shown to be highly accurate compared with alternative approaches like spatial ancestry analysis that, in turn, is slightly more accurate than principal component analysis-based approach for biogeography (Yang et al. 2012; Elhaik et al. 2014). GPS finds the geographical origin of a sample by matching its admixture signature with reference samples of known geographical origin. To infer the geographical coordinates (latitude and longitude) of an individual given *K* admixture proportions, GPS requires a reference population set of *N* populations with both *K* admixture proportions and two geographical coordinates (longitude and latitude). All supervised admixture proportions were calculated as in Elhaik et al. (2014).

Detailed annotation for subpopulations was unavailable for most populations (supplementary fig. S1, Supplementary Material online), though they exhibited fragmented subpopulation structure (fig. 1). To determine the number of subpopulations in each population, we adopted a similar approach to that of Elhaik et al. (2014). Let *Nα* denote the number of samples per population *α*; if *Nα* was less than four individuals, the population was left unchanged. For other populations, we used *k*-means clustering routine with five replications implemented in Matlab. Let *X_ij_* be the admixture proportions of individual *i* in component *j*. For each population, we ran *k*-means clustering for k∈2, using *Nα *×9 matrix of admixture proportions (*X_ij_*) as input. At each iteration, we calculated the ratio of the mean square and sum of squares between the groups. If this ratio was <0.9 and there were more than three samples in each cluster, then we accepted the *k*-component model, whereas smaller clusters were removed.

To bolster the accuracy of GPS inferences beyond what has been previously reported (Elhaik et al. 2014), we have updated the reference panel to comprise highly localized Afro-Eurasian populations. For that, we applied GPS to all Afro-Eurasian individuals (supplementary table S1, Supplementary Material online) using the leave-one-out procedure at the population level. This approach is more rigorous than the leave-one-out individual procedure and ensures that the reference panel will not be biased by outliers that do not fit with the genetic profile of the region. Individuals predicted to reside within the political borders of their countries or <200 km outside of them were retained and were used to recompile the reference population set using the technique described above. This procedure was repeated until the rate of correctly assigned individuals exceeded 80%. Due to their extreme geographical locations Germans and Altai could not satisfy the filtering criteria and were supplemented to the final reference panel using the admixture proportions calculated in a previous round. Overall, we included 26 populations, with some appearing as two subpopulations, in our reference population set (fig. 3). These populations were considered hereafter as *reference populations*.

The geographical distributions of the reference populations (fig. 2*A*) were calculated based on the geographical locations and admixture proportion of the reference populations (fig. 3) using the Matlab function TriScatteredInterp that performs linear interpolation of two dimensional datasets. This allowed us to evaluate the admixture proportion of any coordinate pair within the geographical area covered by the reference populations (fig. 5*D*).

### Calculating the Biogeographical Origin of a Test Sample and Genetic Distances

GPS coordinates for a test individual were calculated as previously described (Elhaik et al. 2014). In brief, given an individual of unknown geographical origin and nine admixture proportions that correspond to nine putative ancestral populations, GPS converts the genetic distances between the test individual and the nearest *M *=**10 reference populations to geographic distances. We defined genetic admixture distance (*d*) as the minimal Euclidean distance between the admixture proportions of an individual to those of all individuals of a certain population. A graph illustrating the genetic distances was plotted using Matlab Graph function.

All maps were plotted using the R package rworldmap (South 2011). The Silk Road and trade route maps were plotted according to the maps available from the Stanford Program on International and Cross-cultural Education (SPICE) interactive resource http://virtuallabs.stanford.edu/silkroad/SilkRoad.html (last accessed March 15, 2016). The geographical coordinates of the Turkish place names were obtained from the Geographical Names website (http://www.geographic.org/geographic_names/, last accessed March 15, 2016).

## Supplementary Material

Supplementary figures S1–S8 and supplementary tables S1–S5 are available at *Genome Biology and Evolution* online (http://www.gbe.oxfordjournals.org/).

Supplementary Data
