# Supplementary material for: Localizing Ashkenazic Jews to Primeval Villages in the Ancient Iranian Lands of Ashkenaz
Source: Genome Biol Evol. 2016 Mar 3;8(4):1132–49. doi: 10.1093/gbe/evw046 (PMC4860683; doi:10.1093/gbe/evw046)
Supplement: Supplementary Data [file supp_8_4_1132__index.html]

Localizing Ashkenazic Jews to Primeval Villages in the Ancient Iranian Lands of Ashkenaz — Supplementary Data 

# Localizing Ashkenazic Jews to Primeval Villages in the Ancient Iranian Lands of Ashkenaz

## Supplementary Data

files

- Supplementary Data - zip file
