# Supplementary material for: Localizing Ashkenazic Jews to Primeval Villages in the Ancient Iranian Lands of Ashkenaz
Source: Genome Biol Evol. 2016 Mar 3;8(4):1132–49. doi: 10.1093/gbe/evw046 (PMC4860683; doi:10.1093/gbe/evw046)
Supplement: Supplementary Data [file supp_evw046_suppl_data.zip › Das et al 2016 - Supp.docx]

Supplementary information

## Figure S1

The genetic distances (*d*) within each reference population.


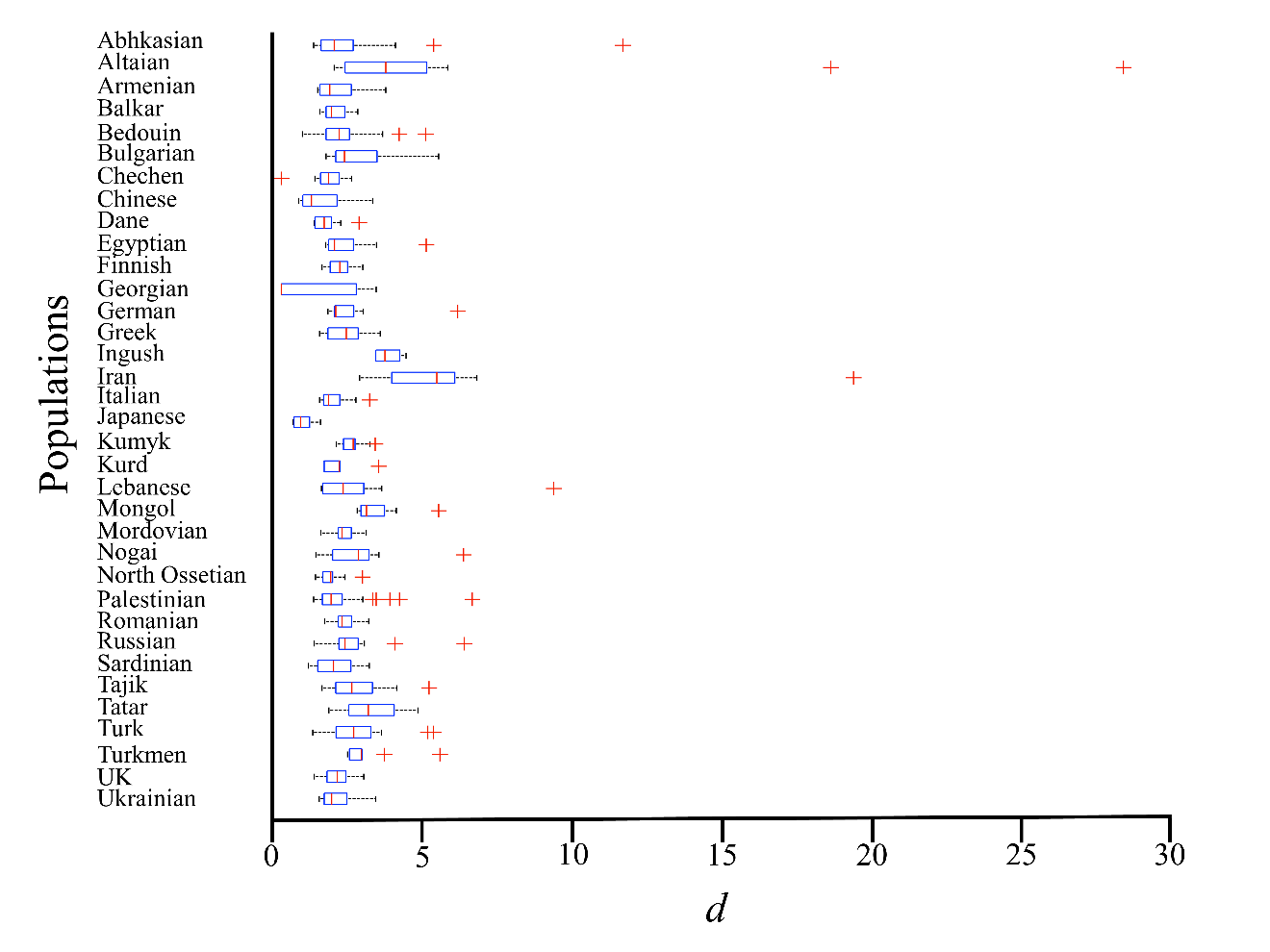


## Figure S2

The genetic distances (*d*) between AJ and their reference populations, where the numbers represent subpopulations identified in our analyses.


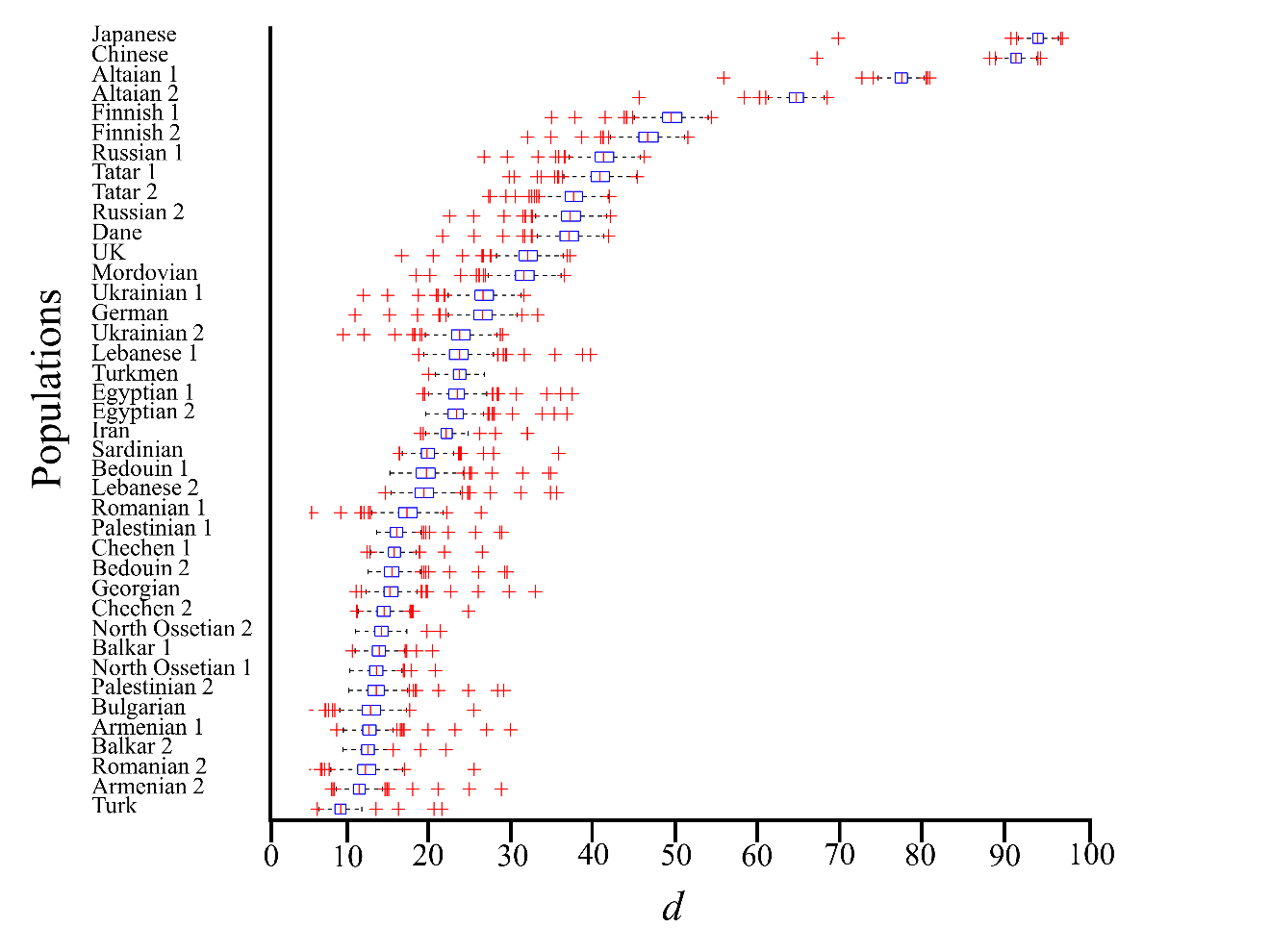


## Figure S3

Admixture proportions of (A) all Ashkenazic Jews, Yiddish (B) and non-Yiddish (C) speakers. The *x*-axis represents individuals from these cohorts. Each individual is represented by a vertical stacked column of color-coded admixture proportions that reflects genetic contributions from nine putative ancestral populations.


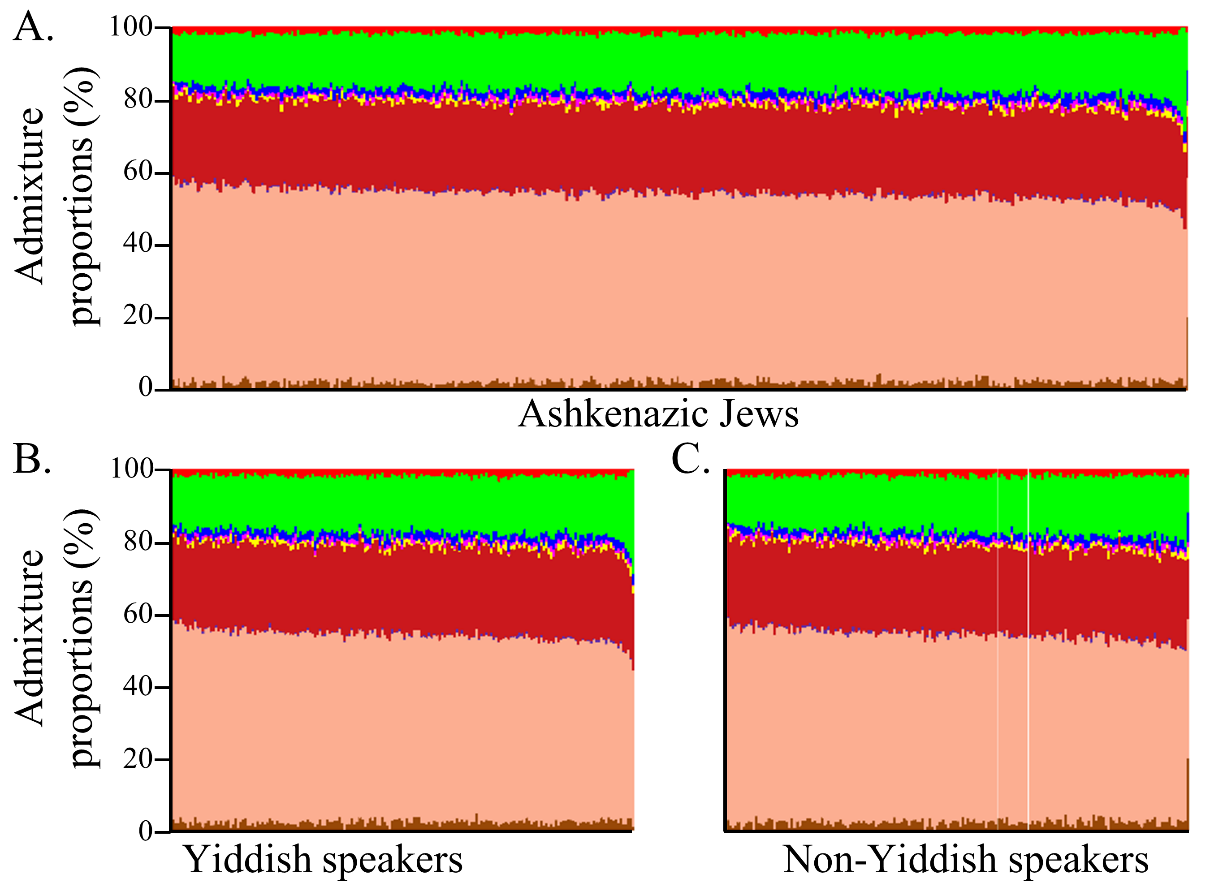


## Figure S4

A map depicting the predicted location of Jewish (triangles) Yiddish speakers (orange), claimants of priestly linages (orange and black), Mountain Jews (pink), and Iranian Jews (yellow) alongside the ancient pre-Scythian individual (blue diamond). An inset shows the sample distribution in northern Turkey ($\tilde{40^{\circ} 41’}$N, $\tilde{38^{\circ}}$E), the locations of the four townships that may derive their names from “Ashkenaz,” and adjacent cities. Large (13-23%), medium (4-10%), and small (1-4%) circles reflect the percentage of Yiddish speakers’ parents born in each region. The paternal and maternal haplogroups of the Yiddish speakers are shown at the top of the figure.


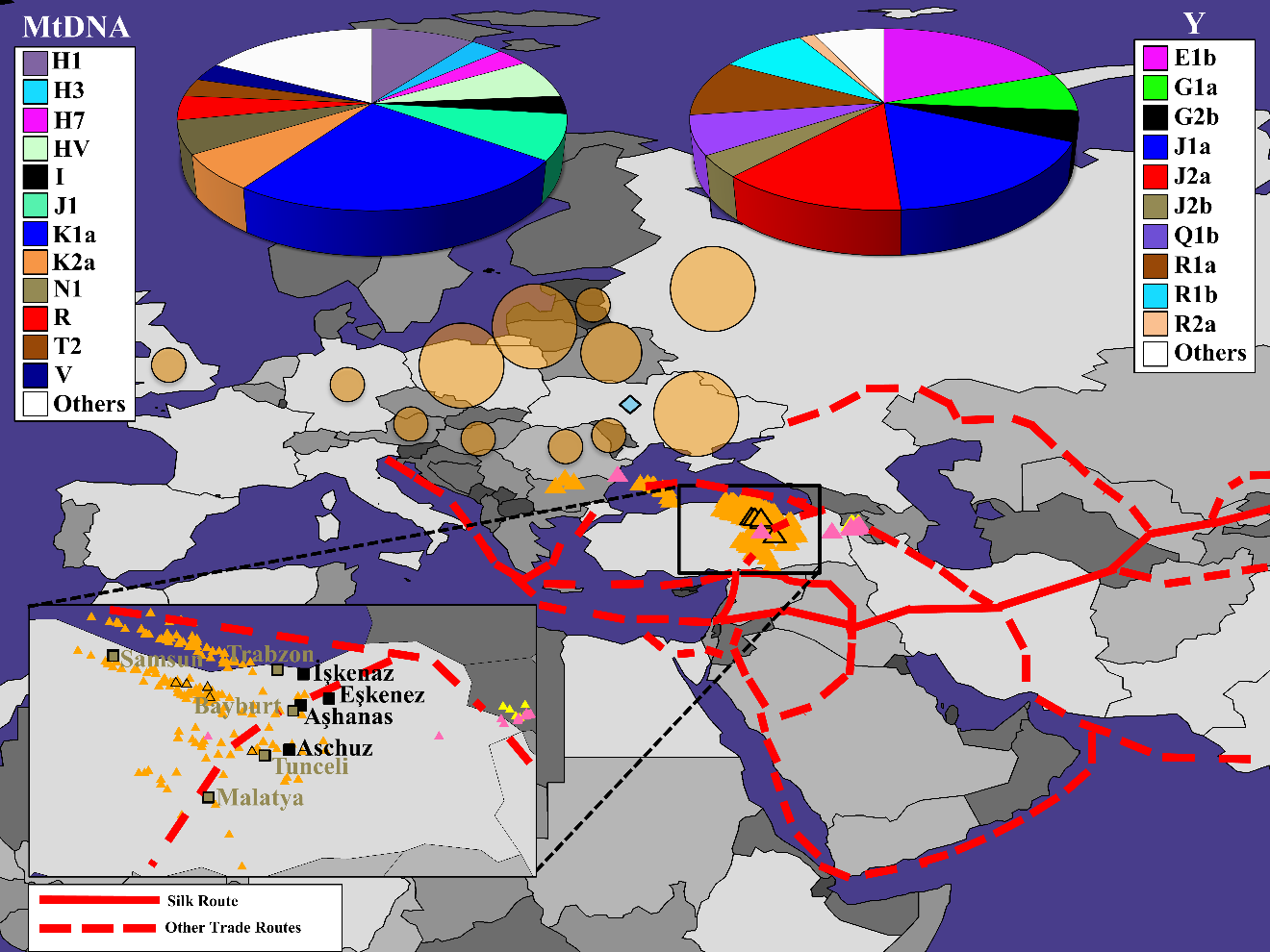


## Figure S5

A map depicting the predicted location of Jewish (triangles) non-Yiddish speakers (orange), claimants of priestly linages (orange and black), Mountain Jews (pink), and Iranian Jews (yellow) alongside the ancient pre-Scythian individual (blue diamond). An inset shows the sample distribution in northern Turkey ($\tilde{40^{\circ} 42’}$N, $\tilde{37^{\circ} 57’}$E), the locations of the four townships that may derive their names from “Ashkenaz,” and adjacent cities. Large (13-23%), medium (4-10%), and small (1-4%) circles reflect the percentage of the non-Yiddish speakers’ parents born in each region. The paternal and maternal haplogroups of the non-Yiddish speakers are shown at the top of the figure.


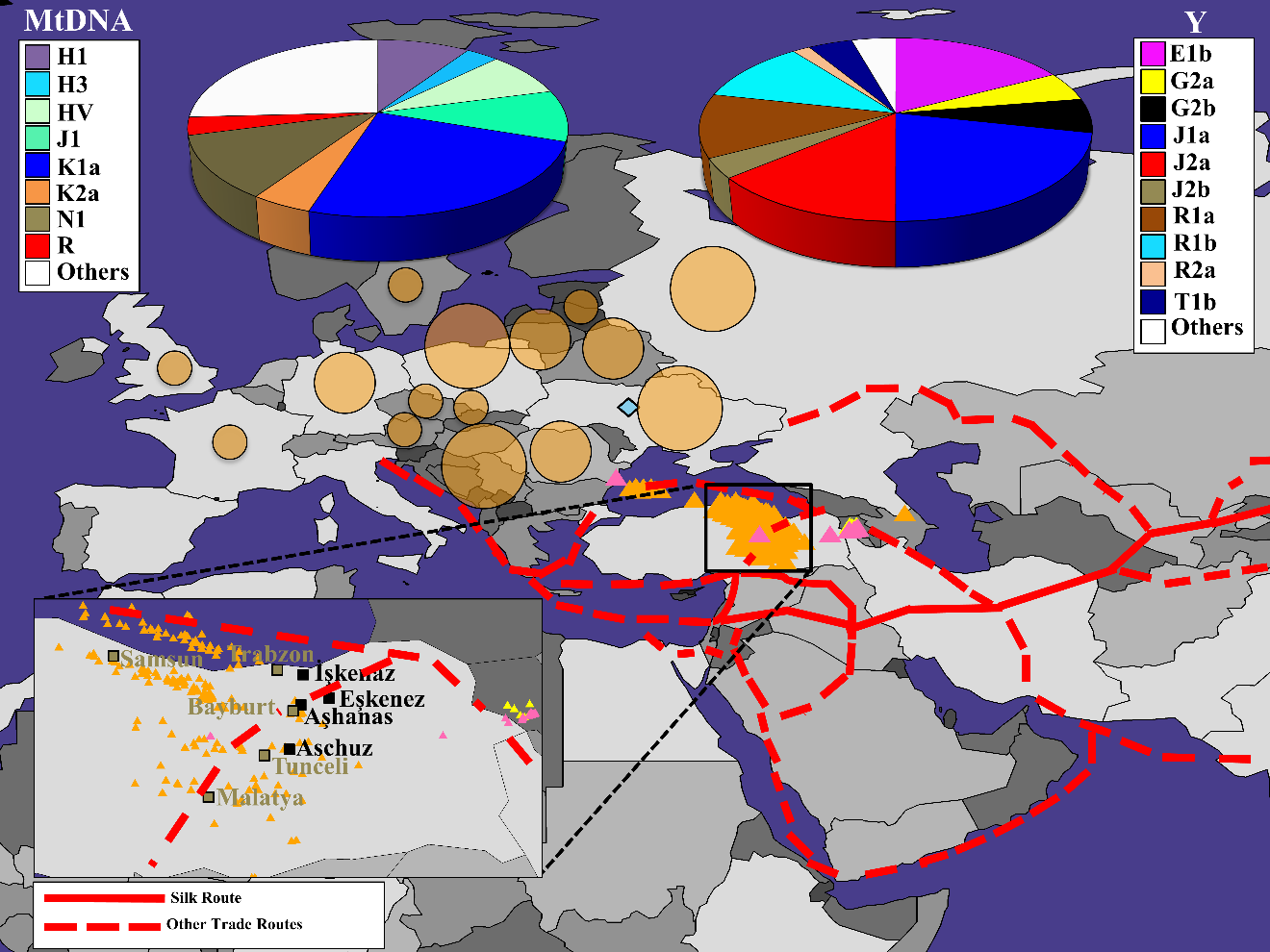


## Figure S6

The population affinities of the most common paternal and maternal haplogroups for Yiddish speakers based on Genographic’s public database. The total number of haplogroups (*n*) and those belong to “Ashkenazic Jews” (*n_AJ_*) are noted.


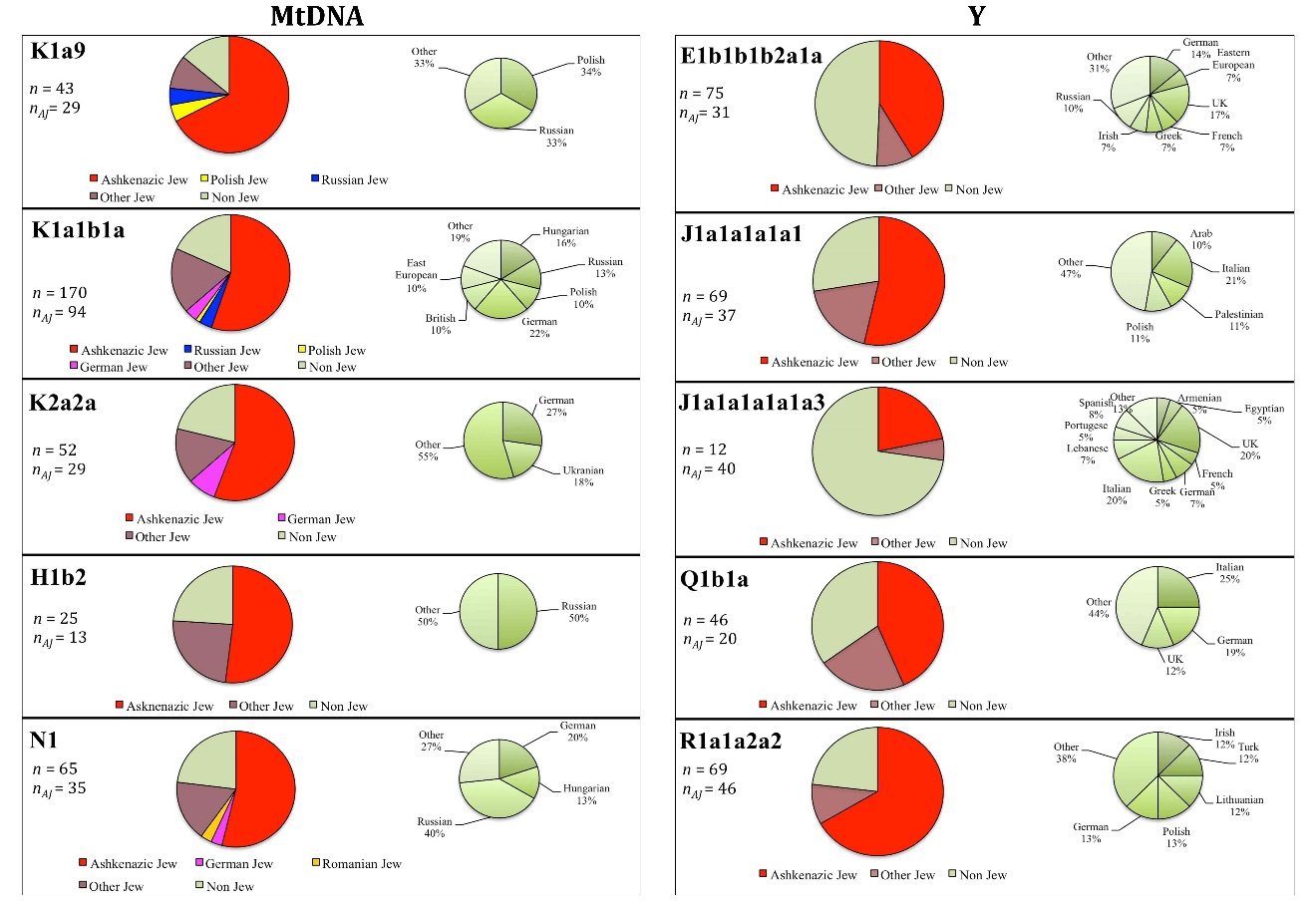


## Figure S7

The genetic distances (*d*) between Yiddish speakers and simulated “native” individuals from six populations. The number of individuals of each region is indicated near the population name.


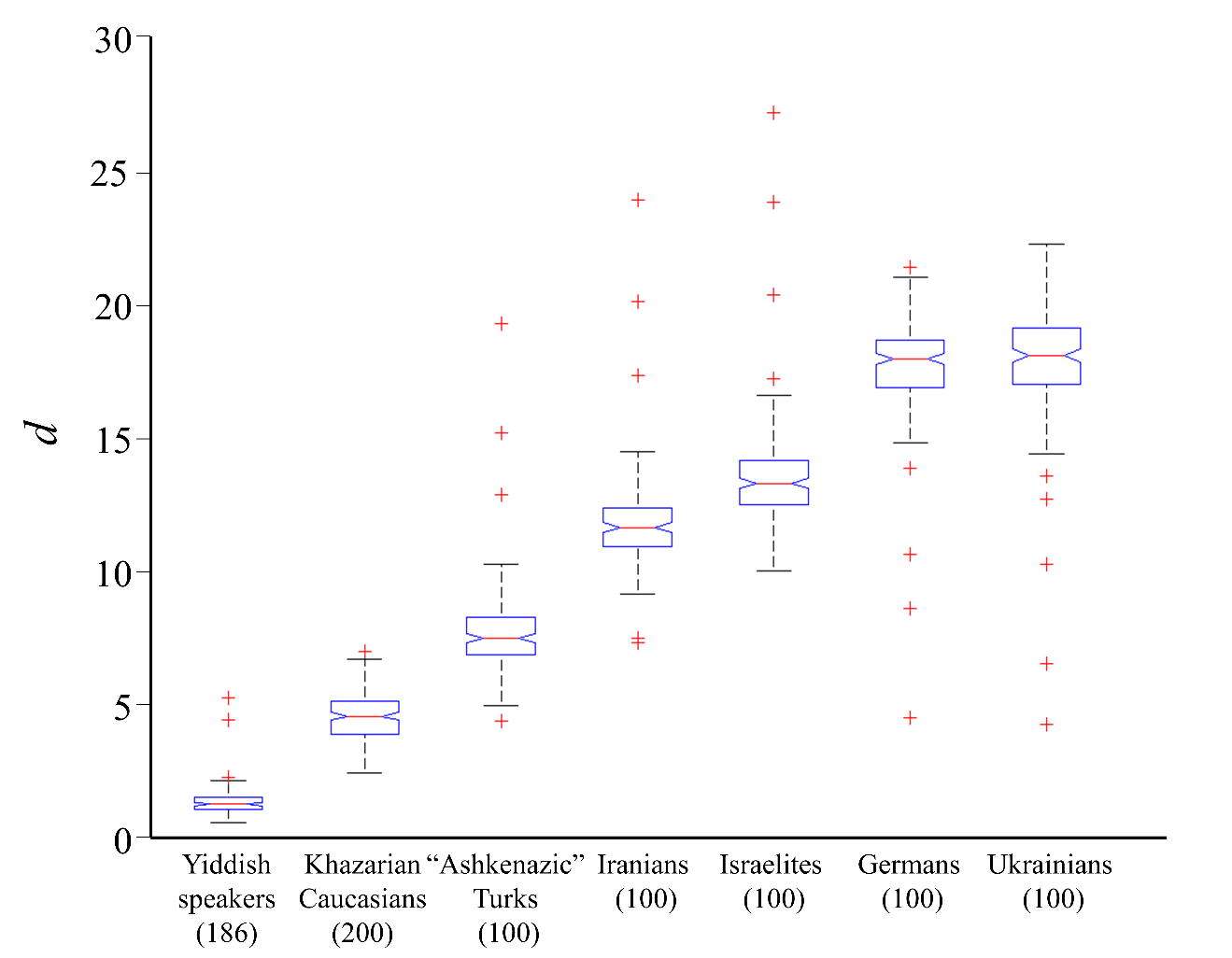


## Figure S8

The genetic distances (*d*) between non-Yiddish speakers and simulated “native” individuals from six populations. The number of individuals of each region is indicated near the population name.


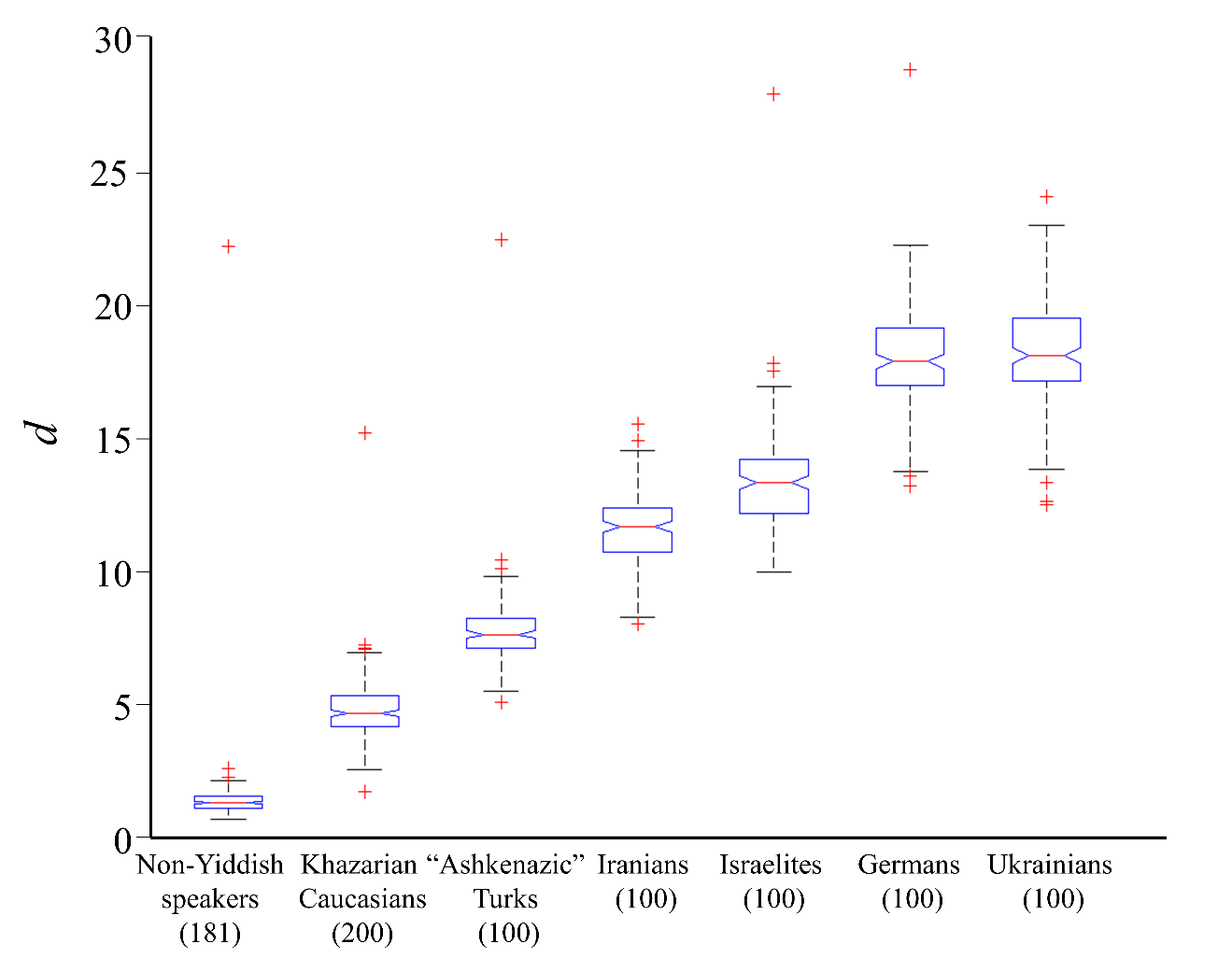


## Table S1 - Summary of reference populations, nicknames, sampling sites, sample sizes (*N*), geographic coordinates, and data sources used for each population

| Populations | Nick | Countries and states/regions | *N* | Latitude | Longitude | Sources |
| --- | --- | --- | --- | --- | --- | --- |
| Abkhazian | AB | Abkhazia | 24 | 42.97 | 41.44 | ^13^ |
| Armenian | ARM | Armenia | 16 | 40.06 | 45.03 | ^1^ |
| Bedouin | BDN | Israel | 45 | 31 | 35 | ^2^ |
| British | UK | United Kingdom | 13 | 52 | -1.4 | ^3^ |
| Bulgarian | BU | Bulgaria | 15 | 42.74 | 25.48 | ^3^ |
| Chinese | CHB | China | 12 | 34.28 | 105.02 | ^3^ |
| Dane | DA | Denmark | 12 | 56 | 10 | ^3^ |
| Egyptian | EG | Egypt | 15 | 30.03 | 31.15 | ^3^ |
| Finn | FIN | Finland | 12 | 60.1 | 24.56 | ^3^ |
| Georgian | GO | Georgia | 7 | 41.43 | 44.47 | ^3^ |
| German | GR | Germany | 13 | 52.51 | 13.38 | ^3^ |
| Greek | GK | Greece | 15 | 37.96 | 23.71 | ^3^ |
| Iranian | IR | Iran | 16 | 32.42 | 53.68 | ^3^ |
| Italian, Sardinian | ID | Italy, Sardinia | 15 | 40 | 9 | ^3^ |
| Italian, Tuscan | TSI | Italy, Tuscan | 15 | 43 | 11 | ^3^ |
| Japanese | JPT | Japan | 12 | 36.02 | 138.25 | ^3^ |
| Kurds | KR | Kazakhstan | 6 | Undefined | | ^1^ |
| Lebanese | LE | Lebanon | 22 | 33.85 | 35.86 | ^3^ |
| Mongolian | MG | Mongolia | 11 | 45 | 111 | ^3^ |
| Palestinian | PAL | Israel | 51 | 32.5 | 35 | ^2^ |
| Romanian | RO | Romania | 15 | 44.8 | 26.06 | ^3^ |
| Russian, Altaian | RA | Russia, Altay | 15 | 50.61 | 86.21 | ^3^ |
| Russian, Balkar | RB | Russia, Balkar | 19 | 43.39 | 43.56 | ^1^ |
| Russian, Chechen | RC | [Russia, Chechnya](http://en.wikipedia.org/wiki/Chechnya) | 26 | 43.4 | 45.71 | ^1,3^ |
| Russian, Ingush | RI | Russia, Chechnya, Ingush | 4 | 43.8 | 45.71 | ^3^ |
| Russian, Kumyk | RK | Russia, Republic of Dagestan | 14 | 42 | 47 | ^1^ |
| Russian, Mordovian | RMO | Russia, Mordvinia | 15 | 54.23 | 44.06 | ^1^ |
| Russian, Moscow | RM | Russia, Moscow | 16 | 55.75 | 37.62 | ^3^ |
| Russian, Nogai | RN | [Russia, Republic of Dagestan](http://en.wikipedia.org/wiki/Ossetia) | 16 | Undefined | | ^1^ |
| Russian, North Ossetian | RNO | [Russia, Ossetia (North)](http://en.wikipedia.org/wiki/Chechnya) | 15 | 43.04 | 44.28 | ^1^ |
| Russian, Tatar | RT | Russia, Tatarstan | 15 | 55.18 | 50.72 | ^3^ |
| Tajikistan | PT | Tajikistan | 28 | 38.35 | 68.48 | ^1,3^ |
| Turk | TUR | Turkey | 19 | 39 | 36 | ^4^ |
| Turkmen | TR | Turkmenistan | 15 | 37.57 | 58.23 | ^1^ |
| Ukrainian | UR | Ukraine | 20 | 50.26 | 30.31 | ^1^ |

## Table S2 - Prediction accuracy for populations and sub populations

|  | Prediction accuracy (%) | |
| --- | --- | --- |
| Populations | <250km | <500km |
| Abkhazian | 88 | 96 |
| Armenian | 100 | 100 |
| Bedouin | 98 | 100 |
| British | 77 | 77 |
| Bulgarian | 100 | 100 |
| Chinese | 42 | 42 |
| Dane | 93 | 93 |
| Egyptian | 100 | 100 |
| Finn | 92 | 92 |
| Georgian | 100 | 100 |
| German | 77 | 77 |
| Greek | 67 | 93 |
| Iranian | 100 | 100 |
| Italian, Sardinian | 100 | 100 |
| Italian, Tuscan | 0 | 0 |
| Japanese | 100 | 100 |
| Kurds | 0 | 0 |
| Lebanese | 77 | 82 |
| Mongolian | 0 | 0 |
| Palestinian | 100 | 100 |
| Romanian | 93 | 100 |
| Russian, Altaian | 0 | 87 |
| Russian, Balkar | 89 | 89 |
| Russian, Chechen | 96 | 100 |
| Russian, Ingush | 100 | 100 |
| Russian, Kumyk | 79 | 100 |
| Russian, Mordovian | 100 | 100 |
| Russian, Moscow | 100 | 100 |
| Russian, Nogai | 0 | 19 |
| Russian, North Ossetian | 100 | 100 |
| Russian, Tatar | 87 | 87 |
| Tajikistan | 0 | 0 |
| Turk | 84 | 84 |
| Turkmen | 100 | 100 |
| Ukrainian | 100 | 100 |

## Table S3 - Demographic data for all Ashkenazic Jews included in this study including coordinates predicted by GPS

Data is reported mostly as provided by the public participants of The Genographic Project.

## Table S4 – mtDNA haplogroup mutations

An exclamation mark indicates back mutation. Parentheses indicates unstable mutation, or more likely, unverified observations.

| **MtDNA HGs** | **Mutations** |
| --- | --- |
| H | G2706A, T7028C |
| H1 | G3010A |
| H10a1b | G513A, T7220C, G10325A, C16344T |
| H11a1 | C8898T, C16278T! |
| H11a2a | T16140C |
| H11b1 | T7645C |
| H13a1a1 | G7337A, T13326C |
| H1ai1 | G6722A |
| H1aj1 | T8618C, C16192T |
| H1aj1a | G207A, G9621A, T16172C, G16456A |
| H1as2 | T980C |
| H1b1 | A3796G |
| H1b1a | 5899.XC, A8348G |
| H1b2a1 | T7691C |
| H1bw | C8478T |
| H1e4 | G3316A |
| H26c | T146C!, A10562G |
| H27 | G11719A!, A16316G |
| H3p | C16222T |
| H3w | C3613T, T5999C, C16248T |
| H40b | G7444A, T7678C |
| H41a | C262T, G5460A, T10124C, A14118G |
| H4a1a3a | G5773A |
| H5~36 | C456T |
| H5a7 | G8557A, C15175T |
| H5c2 | C3819T, G16213A |
| H6a1a1a | A7325G, A9362G, G11611A, T16311C! |
| H6a1a3 | T5785C |
| H6a1a5 | C10936T |
| H7 | A4793G |
| H7c2 | C13959T |
| H7e | A8026t, C9527T |
| HV1a~b~c | A15218G |
| HV1b2 | A3547G, G6023A, T16189C! |
| HV5 | C12133T, A13105G! |
| I1c | G8573A, C16264T, G16319A, T16362C |
| I1c1a | G6267A, A6359G |
| J1b1a1 | T16172C |
| J1c | (G185A), (G228A), T14798C |
| J1c1 | T482C, T3394C |
| J1c14 | T195C!, G4491A, C8940T, A9120G, G16274A, C16355T |
| J1c3b2 | A4829G |
| J1c3e2 | G8865A |
| J1c5 | A5198G |
| J1c7 | C6554T, G12127A, (T16092C) |
| J2a1a1 | A13722G, C16261T |
| J2b1 | G10172A |
| J2b1e | T8843C |
| K1a | C497T, (T16093C) |
| K1a1b1 | A11470G |
| K1a1b1a | A10978G, T12954C, C16234T |
| K1a4a | G6260A |
| K1a9 | A16524G |
| K2a | T152C!, G709A, T4561C |
| K2a2a | C11348T |
| K2a2a1 | A512c, A9254G, G11914A! |
| L2a1 | T182C!, A12693G, T15784C, A16309G |
| L2a1~2~3~4 | C2789T, C7274T, A7771G, G11914A!, A13803G, A14566G, C16294T |
| L2a1l2a1 | C3573a |
| M33 | G2361A |
| N1 | T10238C, G12501A |
| N1~5 | G1719A |
| N1b1b | C4735a, A4917G, A11928G, C12092T, C13129T, A13710G, G16176A |
| R0a2m | A4767G |
| R0a4 | C150T, T2351C, A9531G |
| T1a1 | T9899C |
| T1a1~3 | T195C! |
| T1a5 | T6152C |
| T1b | T16243C |
| T2a1 | A14687G |
| T2b25 | G7521A!, C8934T |
| T2e | C150T, G16153A |
| T2e1b | A9181G |
| T2f | C8270T, 8281-8289d |
| T2f1a1 | C15028a |
| T2g1 | G3834A, A14839G |
| U2e1a1 | C3116T |
| U3a1 | G3010A |
| U5a1b1 | C16291T |
| U5a1b1c2 | G3705A, G16129A! |
| U5a1f1a | T199C, G8251A |
| U5b2a1a | T15511C, C16189T!! |
| U6a7a1b | C150T |
| U7a | C151T |
| U7a5 | 573.XC, G9300A, A13966G, C14245T, G14869c, C16291T, T16304C |
| V15 | C4221T |
| V18 | A508G |
| V1a1 | C5263T |
| V7 | A93G, G7444A |
| V7a | T11899C, G16153A |
| W1 | C7864T |
| W3b1 | G189A!, T7058C |
| X2b7 | T4216C |
| X2e2a | A3948G |

## Table S5 – Y chromosomal haplogroup mutations

The Y haplogroups notation corresponds to 2015 and (2012) trees

| **Y HGs** | **Mutations** |
| --- | --- |
| E1a1 | M44, L632, L634 |
| E1b1b1a1a1a1a | CTS6667, CTS8415 |
| E1b1b1a1b1 | V13, V36, L542, PF2213, PF2214, PF2248, CTS1975, CTS2374, CTS5371, CTS5935, CTS6472, CTS8061, CTS10912, PAGES00102 |
| E1b1b1a1b1a3 | L241 |
| E1b1b1a1b1a3a | F2524 |
| E1b1b1a1c | V22, L677, CTS2548, CTS2817, CTS5479, CTS6434, CTS8892 |
| E1b1b1b1a1a | M183 |
| E1b1b1b2a1a | M34, L787, L797, PF1999, PF2016, PF2018, PF2020, CTS1229, CTS11004 |
| E1b1b1b2a1a3a | CTS1096 |
| E1b1b1b2a1a3a1c | PF6747 |
| E1b1b1b2a1a3a3 | P53 |
| E1b1b1b2a2a | PF1952, PF1974, PF1980, PF1987, PF2010, PF2011, PF2014, PF2026, PF2027, PF2028, PF2031, PF2039, PF2042, PF2046, PF2051, PF2053, PF2057, PF2060, PF2064, PF2068, PF2070, PF2072, PF2073, PF2078, PF2080, PF2082, PF2086, PF2088, PF2089, PF2090, PF6764, PF6766, PF6768 |
| E1b1b1b2a2b | PF3780 |
| E2 | M75, P68, CTS72, CTS132, CTS249, CTS672, CTS714, CTS1173, CTS1191, CTS1248, CTS1633, CTS2239, CTS3092, CTS4310, CTS4406, CTS4847, CTS4912, CTS5333, CTS5477, CTS5920, CTS5938, CTS5989, CTS6510, CTS6651, CTS6731, CTS6881, CTS6951, CTS7139, CTS7215, CTS7550, CTS7684, CTS7804, CTS7864, CTS8138, CTS8331, CTS8550, CTS8850, CTS8923, CTS8958, CTS9016, CTS9348, CTS9424, CTS9452, CTS10020, CTS10246, CTS10361, CTS10680, CTS11086, CTS11130, CTS11207, CTS11318, CTS11446, CTS11447, CTS11524, CTS11817, CTS11867, CTS11960, CTS12035, CTS12049 |
| G1 | M285, M342, F858, F1218, F2447, F2498, F2853, F3062, F4154, F4232, F4262, F4341 |
| G1a | F1761, F2376, F4113, F4297, CTS11562 |
| G1a1b | P37, L201, L202, L203 |
| G2a1a1a | L140, PF3331, PF3337 |
| G2a1a1a1a1a | PF6863, PF6865, PF6866, CTS77, CTS5990 |
| G2a1b1a1 | PF3296, PF3316 |
| G2b1 | M377, L72, L183 |
| I2 | M438, P215, L41, L68, L460, PF3573, PF3595, PF3607, PF3621, PF3623, PF3626, PF3644, PF3651, PF3652, PF3657, PF3658, PF3664, PF3667, PF3669, PF3671, PF3798, PF3801, PF3802, PF3812, PF3820, PF3849, PF3876, CTS3296, CTS4039, CTS5727 |
| J1a1a1 | P58, Z1874, Z1878, Z1890, PF4629, PF4654, PF4663, PF4800, PF4830, PF4831, PF4833, PF4835, PF4844, CTS8437, CTS11501, YSC0000062, YSC0000164, YSC0000181, YSC0000188 |
| J1a1a1a | L147, Z1853, Z1854, Z1855, Z1856, Z1860, Z1865, Z1871, Z1875, Z1885, Z1886, Z1887, Z1889, Z1892, PF4678, PF4799, PF4838, CTS5269, YSC0000071, YSC0000169 |
| J1a1a1a1a1 | YSC0000234 |
| J1a1a1a1a1a | Z1884, PF4851, YSC0000080 |
| J1a1a1a1a1a2 | Z640, Z641, Z644 |
| J1a1a1a1a1a3 | YSC0000076 |
| J1a1a2a | PF7263 |
| J2a1 | F1227, F4074, PF4610, PF5084, PF5091, PF5104, PF5105, CTS7683 |
| J2a1a1b1b1 | PF5366, PF5368 |
| J2a1a1b1b2 | L243 |
| J2a1a1b1c1 | L70, L397, L398, Z386, Z395, Z415, Z425, Z435, Z444, PF5425, PF5441, PF5444, PF7437, CTS359, CTS1486 |
| J2a1a1b1c1a | PF5456, CTS3601, CTS6061 |
| J2a1a2b1a1 | Z515 |
| J2a1a2b1a2a1 | L236 |
| J2a1a2b3b1 | Z482 |
| J2a1a2b3b1a | Z474 |
| J2b1 | Z575, Z593, Z605, Z1827 |
| J2b1a1a | Z590, Z603 |
| J2b1a1b | Z631, Z632, Z634, Z636, Z639, Z1043, Z1048, Z1295 |
| L1a | M317 |
| L1a1a | F168, PAGES00116 |
| Q1b1a | L245 |
| R1a1a1a1 | Z92, CTS456 |
| R1a1a1a2a | CTS3402 |
| R1a1a1b1 | CTS11962 |
| R1a1a2a | Z94 |
| R1a1a2a2 | F1345, F2997, CTS6, CTS3412, CTS3605, CTS8448 |
| R1b1a1a | YSC0000072 |
| R1b1a1a1a | P310, L11, L52, L151, YSC0000082, YSC0000191 |
| R1b1a1a1a1a1 | L21, Z290 |
| R1b1a1a1a1a4 | U152 |
| R1b1a1a1a1a4a | L2 |
| R1b1a1a1a1a4i | L4 |
| R1b1a1a1a1b1a2b1 | Z160 |
| R1b2a | PF6289, PF6327 |
| R2a2a | F1758 |
| R2a4 | L288 |
| T1a1a1b | PAGES00113 |
| T1b1 | L208, CTS931, CTS1818, CTS2611, CTS4085, CTS4916, CTS6000, CTS7169, CTS9163, CTS9506, PAGES00002 |
| T1b1b | Z709 |

**References**

1. Yunusbayev B, Metspalu M, Järve M *et al*: The Caucasus as an asymmetric semipermeable barrier to ancient human migrations. *Mol Biol Evol* 2011; **29:** 359-365.

2. Conrad DF, Jakobsson M, Coop G *et al*: A worldwide survey of haplotype variation and linkage disequilibrium in the human genome. *Nat Genet* 2006; **38:** 1251-1260.

3. Elhaik E, Tatarinova T, Chebotarev D *et al*: Geographic population structure analysis of worldwide human populations infers their biogeographical origins. *Nat Commun* 2014; **5**.

4. Behar DM, Yunusbayev B, Metspalu M *et al*: The genome-wide structure of the Jewish people. *Nature* 2010; **466:** 238-242.
